# Pancreatic Cancer: Molecular Characterization, Clonal Evolution and Cancer Stem Cells

**DOI:** 10.3390/biomedicines5040065

**Published:** 2017-11-18

**Authors:** Elvira Pelosi, Germana Castelli, Ugo Testa

**Affiliations:** Department of Hematology, Oncology and Molecular Medicine, Istituto Superiore di Sanità, Rome 00135, Italy; elvira.pelosi@iss.it (E.P.); germana.castelli@iss.it (G.C.)

**Keywords:** pancreatic ductal adenocarcinoma, cancer stem cells, genomic profiling, membrane cell markers, tumor xenotransplantation assay

## Abstract

Pancreatic Ductal Adenocarcinoma (PDAC) is the fourth most common cause of cancer-related death and is the most lethal of common malignancies with a five-year survival rate of <10%. PDAC arises from different types of non-invasive precursor lesions: intraductal papillary mucinous neoplasms, mucinous cystic neoplasms and pancreatic intraepithelial neoplasia. The genetic landscape of PDAC is characterized by the presence of four frequently-mutated genes: *KRAS*, *CDKN2A*, *TP53* and *SMAD4*. The development of mouse models of PDAC has greatly contributed to the understanding of the molecular and cellular mechanisms through which driver genes contribute to pancreatic cancer development. Particularly, oncogenic KRAS-driven genetically-engineered mouse models that phenotypically and genetically recapitulate human pancreatic cancer have clarified the mechanisms through which various mutated genes act in neoplasia induction and progression and have led to identifying the possible cellular origin of these neoplasias. Patient-derived xenografts are increasingly used for preclinical studies and for the development of personalized medicine strategies. The studies of the purification and characterization of pancreatic cancer stem cells have suggested that a minority cell population is responsible for initiation and maintenance of pancreatic adenocarcinomas. The study of these cells could contribute to the identification and clinical development of more efficacious drug treatments.

## 1. Introduction

The pancreas possesses two functional cellular compartments, endocrine and exocrine. The exocrine pancreas comprises acinar, ductal and centroacinar cells. An undifferentiated pancreatic trunk epithelium, called pancreatic cords, is present during early stages of embryonic development; these pancreatic cord cells proliferate and differentiate into endocrine and exocrine lineages. Ductal cells, usually quiescent in the adult pancreas, form an intricate network of ducts representing the conduit for the flow of digestive enzymes secreted by acinar cells. Acinar cells are responsible for the secretion of digestive enzymes and represent the preponderant cell type in the pancreas; these cells have an intrinsic plasticity in that they have the capacity to undergo metaplasia to ductal or ductal-like cells: this metaplastic process is known as acinar-ductal metaplasia, occurs during acute-chronic pancreatitis and may represent an initial step towards the pancreatic intraepithelial neoplasia formation [1].

Malignant neoplasms of the pancreas are currently classified based on their cellular differentiation (ductal, acinar or neuroendocrine) of the neoplastic cells, combined with the macroscopic appearance (solid or cystic) of the tumors. Pancreatic ductal adenocarcinoma comprises about 90% of all malignant pancreatic neoplasms. Of all other malignant pancreatic neoplasms (pancreatic neuroendocrine tumors, solid-pseudo papillary neoplasm, acinar cell carcinoma and pancreatoblastoma), neuroendocrine tumors are the most common, comprising approximately 5% of malignant pancreatic tumors [2]. The main features of pancreatic tumors are summarized in Table 1.

Pancreatic adenocarcinoma is a highly aggressive cancer with more than 330,000 estimated deaths in the world in 2012, ranking as the seventh leading cause of cancer death, and is the most lethal of the common malignancies [3]. For 2017, the National Cancer Institute estimates that pancreatic cancer will represent 3.2% of all cancers and 7.2% of all cancer deaths. The incidence of pancreatic cancer has been continuously rising during the last few years, and the projected incidence and death to 2030 predict that pancreas cancer will be the second cause of death by 2030 [4]. The risk of developing pancreatic cancer is about three-times higher in smokers than in nonsmokers, and 5–10% of patients with pancreatic cancer have a family history of the disease. This tumor is characterized by early spread with local diffusion and metastasis to distant organs, and most patients arrive at the clinical diagnosis with surgical unresectable disease: in fact, about 80% of patients are diagnosed with locally advanced or metastatic disease. These patients have a rapid disease progression, and few of them survive more than one year. Even for patients with localized disease at diagnosis and undergoing radical curative surgery, the median survival remains low, being around 18 months [2,5]. Therefore, despite the advances in the understanding of the basic biology of pancreatic adenocarcinoma, survival rates remained basically unmodified during the last 30 years. Therefore, there is an absolute need for the development of new therapeutic strategies for pancreatic adenocarcinoma.

Infiltrating ductal carcinoma of the pancreas arises from different types of non-invasive precursor lesions. Three different types of precursor lesions have been identified: intraductal papillary mucinous neoplasms (IPMNs), mucinous cystic neoplasms (MCNs) and pancreatic intraepithelial neoplasia (PanIN). These precursor lesions meet all the criteria to be really considered as precancerous lesions, precursor to invasive cancer. MCNs are defined as mucin-producing cyst-forming epithelial neoplasms of the pancreas with a peculiar stromal tissue. IPMNs are non-invasive mucin-producing epithelial tumors, usually giving rise to the formation of long finger-like papillae. PanINs are non-invasive small epithelial tumors, located in the smaller pancreatic ducts, and characterized by cytological and architectural atypia [6]. Pancreatic intraepithelial neoplasias are classified into different stages according to the degree of histologic abnormalities in the ductal epithelium. In fact, these neoplasias range from PanIN1A and PanIN-1B to PanIN-2 and PanIN-3, with each stage showing increased cellular and nuclear atypia’s. PanIN-3 is frequently observed in association with invasive pancreatic cancer and seemingly represents a precursor lesion to invasive disease.

It is important to note that a typical feature of pancreatic cancer consists of the formation of a dense stroma, termed a desmoplastic reaction, composed of cellular and fibrillary elements. Pancreatic stellate cells play a key role in the genesis of desmoplastic reaction through their activation of Transforming growth factor β1 (TGF-β1), Fibroblast Growth Factor (FGF), Platelet-derived growth factor (PDGF) and their differentiation into myofibroblasts that actively secrete collagen and other components of the extracellular matrix. The desmoplastic reaction plays a key role in tumor formation, progression, invasion and metastasis. A peculiarity of the pancreatic cancer stroma is given by its capacity to contribute to creating an immunosuppressive tumor microenvironment that restricts antitumor immunity. Recent studies showed that pancreatic stellate cells play also a key role as modifiers of the pancreatic tumor metabolism through the secretion of non-essential amino acids and, particularly, of alanine, which outcompetes glucose and glutamine-derived carbon to fuel the tricarboxylic acid (TCA) cycle [7]. This metabolic change triggered by stellate cells decreases the tumor’s dependence on glucose and serum-derived nutrients [7].

## 2. Genetic Abnormalities

**Figure 1:**
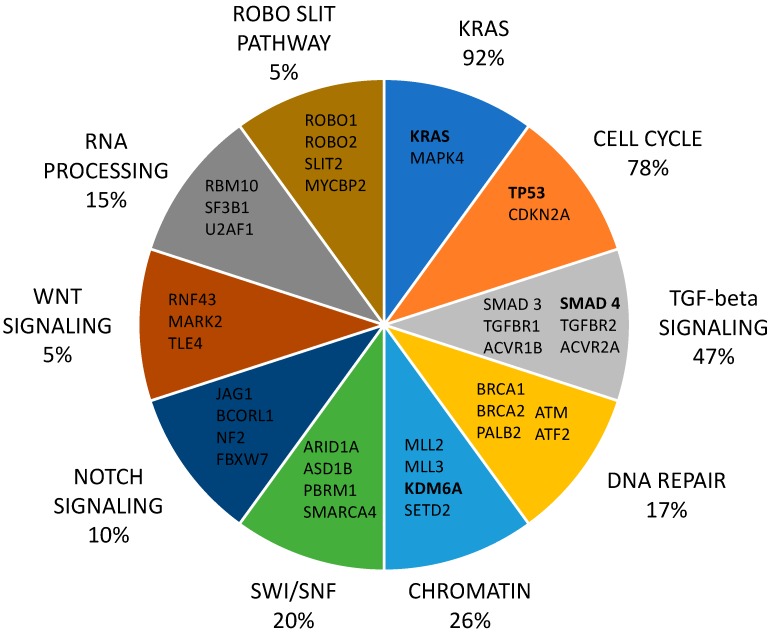
Mutational landscape of pancreatic ductal cancer. The genetic alterations are subdivided into various biochemical pathways, and their global frequency is reported. The most frequent and relevant drivers of pancreatic tumorigenesis are indicated in bold.

The large majority of pancreatic cancers are represented by infiltrating ductal adenocarcinomas. These tumors at the microscopic level are composed by an infiltrating gland-forming neoplastic epithelium, associated with an intense desmoplastic reaction, this reaction being usually very intense. A fundamental step in our understanding of pancreatic adenocarcinoma is represented by the discovery of the main genetic somatic abnormalities observed in this tumor. In this context, considerable progresses have been achieved during the last few years. In 2008, the complete analysis of the pancreatic cancer exome has been reported [8]: the coding regions of >29,000 genes were sequenced in pancreatic adenocarcinomas, showing an average of 63 genomic alterations, in large majority point mutations. These alterations defined a core set of 12 cellular signaling pathways and processes that were altered in the majority (from 67–100%) of pancreatic adenocarcinomas: among them, the most notable were KRAS signaling, regulation of the G_1_/S cell cycle transition, TGF-β signaling, integrin signaling, regulation of cell invasion, homophilic cell adhesion and small guanine triphosphate (GTPase)-dependent signaling [8] (Figure 1).

The genetic landscape of pancreatic tumor genomes is characterized by the presence of four frequently-mutated genes, represented by: *KRAS* (90%), *CDKN2A* (*p16*, 90%), *TP53* (70%) and *SMAD4* (55%). Transcription of the mutant KRAS gene determines the production of an abnormal, constitutively-activated RAS protein, determining as a consequence the uncontrolled activation of proliferation and survival pathways. On the other hand, the frequent inactivation of the *CDKN2A* gene results in a loss of p16 protein, a master negative regulator of the G_1_-to-S transition of the cell cycle, with consequent stimulation of the proliferative activity. The frequent TP53 inactivation permits the cells to bypass some important control checkpoints at the level of DNA damage and apoptotic triggering. Finally, the frequent loss of *SMAD4* gene results in the aberrant signaling by TGF-β.

The mutational status of the most frequently-mutated genes in pancreatic carcinoma was correlated with survival, and it was found that *SMAD4* mutations were associated with poorer prognosis and an increased propensity to metastasize [9]. Recent studies have confirmed that inactivation of *SMAD4* is the strongest predictor of metastatic recurrence [10]. Furthermore, review and meta-analysis of the literature data confirmed the negative clinicopathological significance of *SMAD4* loss in PDACs [11]. Although *SMAD4* gene deletion is associated with a poor prognosis, however, it exposes PDAC cells to a metabolic vulnerability. In fact, Dey and coworkers have analyzed PDACs that harbor the abortion of both copies of the *SMAD4* gene and showed that this subset of tumors is associated with the loss of neighboring genes involved in key metabolic pathways, including mitochondrial malic enzyme 2 (*ME2*) [12]. The ME2 loss in these cells determines a compensatory increased expression of the closely related mitochondrial malic enzyme 3 (*ME3*) [12]. ME2 and ME3 are two enzymes involved in the conversion of malate to pyruvate, and PDAC cells with biallelic *SMAD4* loss are vulnerable to M3 loss or inhibition [12].

Recently, a new exome sequencing and copy number analysis on a large cohort (142 patients) of pancreatic adenocarcinomas has been reported [10]. The results of this analysis allowed defining 16 significantly mutated genes, including the four driver/founder genes *KRAS*, *TP53*, *CDKN2A* and *SMAD4*, other genes already known to be mutated in pancreatic cancer such as *MLL3*, *TGFBR2*, *ARID1A* and *SF3B1* and unreported novel mutant genes, such as genes involved in chromatin modification (*EPC1* and *ARID2*) and DNA damage repair (*ZIM2, MAP2K4, NALCN, SLC16A4* and *MAGE/A6*) [13]. In addition, these authors identified frequent and various somatic genetic alterations of genes classically classified as embryonic regulators of axon guidance, particularly SLIT/ROBO signaling, thus suggesting that this pathway could play a role in pancreatic carcinogenesis [13].

It is important to note that among these mutated genes, no frequent drug targets were found that could be counterbalanced with specific drugs. A notable exception was represented by the identification of rare mutations, such as the mutation of the *PALB2* gene found in one individual cancer, predicting a high sensitivity to DNA damaging agents: in line with this prediction, the treatment of this patient with alkylating agents resulted in marked tumor regression and long-term survival [14].

These four genes (*KRAS*, *TP53*, *CDKN2A* and *SMAD4*) are considered as driver genes for pancreatic adenocarcinoma. In some patients, the mutations present in the primary tumor and in the corresponding metastases have been analyzed. Through this comparative analysis, two categories of mutations were identified: (a) the largest category of mutations corresponded to those present both in primary tumor and in their corresponding metastases (these mutations range from 48–83% of all mutations); (b) the other mutations (these mutations range from 17–51% of all mutations) are defined as progressor mutations and were present in the metastases, but not in the corresponding primary tumors [10]. Importantly, the driver mutations at the level of the genes encoding *KRAS*, *TP53*, *SMAD4* and *CDKN2A* were already present in the primary tumors and remained present at the level of metastases [15]. According to these observations, the driver mutations must be regarded as the founder mutations also [15].

Analysis of genetic abnormalities acquired in metastases showed that during disease progression pancreatic cancer acquires rearrangements indicative of telomere dysfunction and abnormal cell-cycle control, particularly dysregulated G_1_-to-S phase transition [16].

The involvement of abnormalities of genes involved in chromatin remodeling was carefully assessed in a recent study of high-resolution genomic profiling of pancreatic cancer, integrated with mutational data [17]. This analysis allowed identifying structural alterations at the level of multiple subunits of the switch/sucrose non-fermentable (SWI/SNF) chromatin remodelers [17]. In fact, Shain et al. identified somatic genetic abnormalities (genomic deletions, mutations and rearrangements) occurring at the level of genes encoding components of the SWI/SNF chromatin remodeling complex, involving the DNA binding subunits ARID1A, ARID1B and PBMR1 and the enzymatic subunits SMARCA2 and SMARCA4 [17]. Although the frequency of mutation of each of these genes occurred at relatively modest frequency, the global incidence of all these mutations is considerable since hey affected about 1/3 of all pancreatic cancers [17].

Two recent studies contributed to a better definition of molecular abnormalities underlying pancreatic cancer and provided a definition of the genetic heterogeneity of this disease (Figure 1). Thus, a first study performed deep genome sequencing of 100 pancreatic adenocarcinomas, providing evidence that variation in chromosomal structure is a key mechanism underlying DNA damage in pancreatic cancer development [18]. Chromosomal rearrangements determining events of gene disruption were prevalent and recurrently affect some genes, such as *TP53*, *SMAD4*, *CDKN2A*, *ARID1A* and *ROBO2*, as well as *KDM6A* and *PREX2* [18]. The pattern of chromosomal structural variation allowed the classification of ductal pancreatic adenocarcinomas in four groups: a stable subtype (20% of total), containing ≤50% structural variation events and often exhibiting extended aneuploidy and with a frequency of *TP53*, *SMAD4* and *KRAS* mutation in the range; a locally rearranged subtype (30% of total), exhibiting a significant focal event on one or two chromosomes and often displaying focal amplifications in copy number gains harboring known oncogenes, such as *KRAS*, *SOX9*, *GATA6*, *ERB2*, *MET*, *CDK6*, *PI3KCA* and, in some cases, involving complex genomic events, including chromothripsis and breakage-fusion-bridge; a scattered subtype (36% of total), exhibiting a moderate range of non-random chromosomal damage and ≤200 structural variation events; an unstable subtype (14% of total) exhibiting a high level of structural variation events (>200), seemingly related to defects in DNA maintenance; importantly, the unstable subtype was associated with a marked sensitivity to platinum-based therapies [18]. A second study provided the results of an integrated genomic analysis of a large set (456) of PDACs, showing the identification of 32 recurrently mutated genes that make part of 10 different biochemical pathways, including KRAS (92%), TGF-β signaling (47%), NOTCH, WNT (*RNF43* in 5%), ROBO/SLIT signaling, G_1_-to-S transition (*TP53*, *CDKN2A* in 78%), SWI-SNF (*ARID1A* and *SMATCA4*, in 14%) and chromatin modification (*KDM6A*, *SETD2* in 24%) [19] (Figure 1). Expression analysis studies provided evidence that PDACs can be classified into four groups, preferentially associated with specific histologic characteristics: squamous (preferentially associated with adenosquamous carcinomas); pancreatic progenitor; immunogenic (preferentially associated with mucinous non-cystic adenocarcinomas and mucinous carcinomas); aberrantly differentiated-endocrine exocrine (ADEX, preferentially associated with acinar cell carcinomas). The gene expression pattern of squamous subtype tumors is characterized by preferential expression of pathways involved in inflammation, hypoxia response, metabolic reprogramming, TGF-β signaling, MYC pathway activation, autophagy and upregulation of TP63 Delta-N (TP63∆N) and its target genes. The squamous subtype is preferentially associated with *TP53* and *KDM6A* mutations; high TP63 expression is a typical feature of the squamous subtype [19]. The pancreatic progenitor subtype is particularly enriched in transcriptional networks characterized by the transcription factors PDX1, MNX1, HNF4G, HNF4A, HNF1B, HNF1A, FOXA2, FOXA3 and HES1 typically expressed and defining pancreatic progenitors [19]; these tumors are characterized by the expression of some apomucins (MUC1, MUC2 and MUC6) and by frequent TGFBR2 inactivating mutations [19]. The DAEX subtype represents a subclass of pancreatic progenitor tumors and is characterized by a transcriptional network involving transcription factors involved in acinar cell differentiation and genes involved in endocrine differentiation [19]. The immunogenic subtype shares many properties with the progenitor subtype, but differs from this one by the association with a significant immune infiltrate, and gene expression characterized by the expression of B-cell and T-cell signaling pathways [19]. Three of the four PDAC subtypes overlap with the Collisson classification, except for the immunogenic subtype: thus, the pancreatic progenitor subtype corresponds to the classical subtype, the ADEX to the exocrine-like and the squamous to the quasi-mesenchymal subtype [20].

It is important to note that oncogenic *KRAS* mutations that are present in >90% of pancreatic adenocarcinomas are already detectable in precursor lesions, including early preinvasive intraepithelial neoplasia. The accumulation of additional inactivating driver mutations at the level of *P16/CKN2A*, *TP53* and *SMAD4* genes occurs with high frequency at later stages of development on intraepithelial neoplasia. These mutations are required for the progression to invasive pancreatic adenocarcinoma.

A recent study reported an integrated genomic, transcriptomic and proteomic profiling of 150 PDACs. Deep whole-exome sequencing showed recurrent somatic mutations in *KRAS*, *TP53*, *CDKN2A*, *SMAD4*, *RNF43*, *ARID1A*, *TGFBR2*, *GNAS*, *RREB1* and *PBRM1*. Among these various mutated genes, the *RREB1* gene was reported for the first time to be recurrently mutated (5%) in PDACs [15]. RREB1 is activated by the MAPK pathway, represses the miR-143/145 promoter and is downregulated in PDACs. This study provided a characterization of the rare cases of PDACs *KRAS* wild-type (7% of total); these tumors displayed mutations in other drivers, such as *GNAS*, *JAK1*, *CTNNB1*, *BRAF* and showed significantly elevated TSC/MTOR signaling activity, compared to the KRAS-mutant tumors [21]. The type of analysis of *KRAS* mutations showed that the G12D mutant was the most frequent (48%), followed by G12V (31%) and G12R (21%); interestingly, 4% of PDACs display multiple *KRAS* mutations (some showing evidence of biallelic mutations), and these different *KRAS* mutations occurred in separate neoplastic cells in a single tumor [21]. Integrated analysis of the DNA methylation and mRNA expression data showed that 98 genes were silenced by DNA methylation, including genes that have been involved in the development of cancers, such as *ZFP82*, *PARP6*, *BRCA1* and *MGMT* [21]. Finally, protein profiling identified a favorable prognosis subset with low epithelial to mesenchymal transition and high MTOR pathway scores [21].

The presence in the tumor of mutations of one of these founder genes or, concomitantly, in the same tumor of more than one of these genes has key prognostic implications. In fact, Yachida and coworkers provided evidence that the number of driver altered genes was significantly correlated with the overall survival and the disease-free survival [22]. Thirty seven percent of patients had an alteration in all four genes (*KRAS*, *TP53*, *CDKN2A* and *SMAD4*). Carcinomas with only one or two mutations are enriched for these patients with the longest survival [22]. Another recent study confirmed the prognostic impact of the mutational status of founder genes [23]. Abnormal immunolabeling of TP53 was observed in 81% of pancreatic cancer patients and was associated with tumor dedifferentiation and loco regional recurrence; loss of p16 immunolabeling was observed in 67% of patients and was associated with lymphatic invasion and postoperative widespread metastases; loss of SMAD4 immunolabeling was observed in 60% of patients and was associated with tumor size, lymph node metastasis and lymphatic invasion [23]. Very interestingly, all patients surviving at five or more years displayed intact SMAD4 [23].

As reported above, the large majority of pancreatic cancers display *KRAS* mutations. Interestingly, a small subset of patients (1.5%) displays *BRAF* alterations mutually exclusive with *KRAS* mutations [24]. It is of interest to note that not all pancreatic cancers have a mutation of either *KRAS* or *BRAF*: thus, about 5% of these tumors do not possess an RAS mutation. As above stated, the *CDKN2A* locus is inactivated in >90% of pancreatic cancers. This locus encodes for both p16^INK4A^ and p19^ARF^, and the current evidence indicates that both proteins are inactivated by large gene deletions occurring in pancreatic cancer. Interestingly, in a subset of pancreatic cancers retaining *CDKN2A*, somatic mutations of other cell-cycle regulators such as *FBXW7* or *ANAPC2* occur. *TP53* is mutated in up to 85% of pancreatic cancer through various molecular mechanisms involving nonsense mutations, frameshifts and homozygous deletions. In pancreatic cancer not displaying *TP53* mutations, alterations of other genes provide a mechanism of inactivation of one or more p53 functions.

In addition to these studies focused on analyzing the mutational spectrum of pancreas carcinoma advanced lesions, other studies addressed the investigation of gene mutations in tumor precursor lesions (Table 1). Thus, Hong and coworkers have reported the genome-wide analysis of PanIN lesions. To perform this analysis, these authors have explored PanINs and IPMNs in patients with a family history of pancreatic cancer [25]. The results of this interesting study showed that more than 80% of PanIN and IPMN lesions do not have common somatic copy number gene alterations; in contrast, about 95% of PanINs and IPMNs harbor mutations in *KRAS* [25]. According to these findings, it was proposed that: (a) familial precursor lesions of pancreatic adenocarcinoma do not start through the inactivation of a tumor suppressor gene; (b) *KRAS* mutations usually precede tumor suppressor gene inactivation in precursor lesions; (c) among the precursor lesions that displayed copy number alterations, there is no one common tumor suppressor locus targeted [25]. The lower grade lesions PanIN-1 and PanIN-2 often harbor genetic alterations at the level of KRAS and *CDKN2A* genes, while the higher-grade Pan-IN3 lesions display, in addition to these two mutations, also TP53 and *SMAD4* gene mutations [26]. However, Hosoda and coworkers failed to find SMAD4 mutations in high-grade PanINs [27], while they reported the frequent occurrence of *KRAS* (94%), *CDKN2A* (35%) and *RNF43* (30%), while mutations in *TP53*, *GNAS*, *ARID1A*, *PIK3CA* and *TGFBR2* are only rarely observed [27]. Whole exome sequencing of five high-grade PanIN showed several somatic mutations ranging from 30–175 [27].

Another recent study reported the identification of a gene frequently observed to be mutated in IPMNs. Thus, Wu and coworkers reported the mutation of the oncogene GNAS occurring in about 66% of IPMNs: importantly, in the large majority of IPMNs progressing to PDAs with GNAS mutations, the mutation of this gene was observed also at the level of the invasive carcinoma [27]. Interestingly, *GNAS* mutations were not found in other types of cystic neoplasms of the pancreas or in invasive carcinomas not associated with previous IPMN lesions [19]. These observations suggest that *GNAS* mutations define a pathway for pancreatic cyst and adenocarcinoma development [28].

Other studies have shown that components of ubiquitin-dependent pathways are frequently mutated in pancreatic cancer precursor lesions. These findings were observed in the context of a study aiming to analyze the genomic landscapes of neoplastic cysts of the pancreas. Somatic genetic mutations were more frequent in IPMNs than in other cystic neoplasms. Among the various mutations identified, mutations of the E3 ubiquitin ligase components were particularly relevant. In fact, 50% of serous cystadenomas contained mutations of the VHL gene; interestingly, 75% of IPMNs and about 35% of mucinous cystic neoplasms (MCNs) contained mutations of *RNF43*, a gene encoding for a protein with intrinsic E3 ubiquitin ligase activity, thus suggesting that this may act as a suppressor of IPMNs and MCNs [29].

A recent study reported the molecular characterization of a large set of IPMNs showing that 91% of these tumors display *KRAS* or *GNAS* mutations and 47% had mutations of both genes; mutations of RNF43 are observed in 38% of cases; *CDKN2A* (3%), *CTNNB1* (6%), *SMAD4* (5%), *TP53* (9%) mutations are rare; copy number alteration events are less frequent and involve genes such as *RNF43* (11%), *SMAD4* (10%), *CDKN2A* (8%), *VHL* (4%); finally, aneuploidy was observed in 50% of samples [30].

Pancreatic acinar cells can de-differentiate to a progenitor phenotype that expresses ductal markers, through a process collectively known as acinar-to-ductal metaplasia (ADM) (Table 1). ADM is a process commonly observed during pancreatitis and contributes to the regeneration of acinar structures and reconstitution of the pancreas [31,32]. Although ADM was shown to represent an initiating event for development of pancreatic cancer in mice, there is no proof that ADM contributes to PDAC development in humans. Attempts have been made to explore the occurrence and the significance of DAM in human PDACs. Analysis of ADM human lesions for KRAS mutations showed that ADM associated with PanIN harbored the same KRAS mutations, while those not associated with PanIN were KRAS wild-type [33]. According to these findings, it was concluded that ADM lesions are genetically distinct from PanIN, and those in association with PanIN may represent retrograde extension of the PanIN [33]. Thus, it seemed unlikely that ADMs are precursors to PanINs [33].

**Figure 2:**
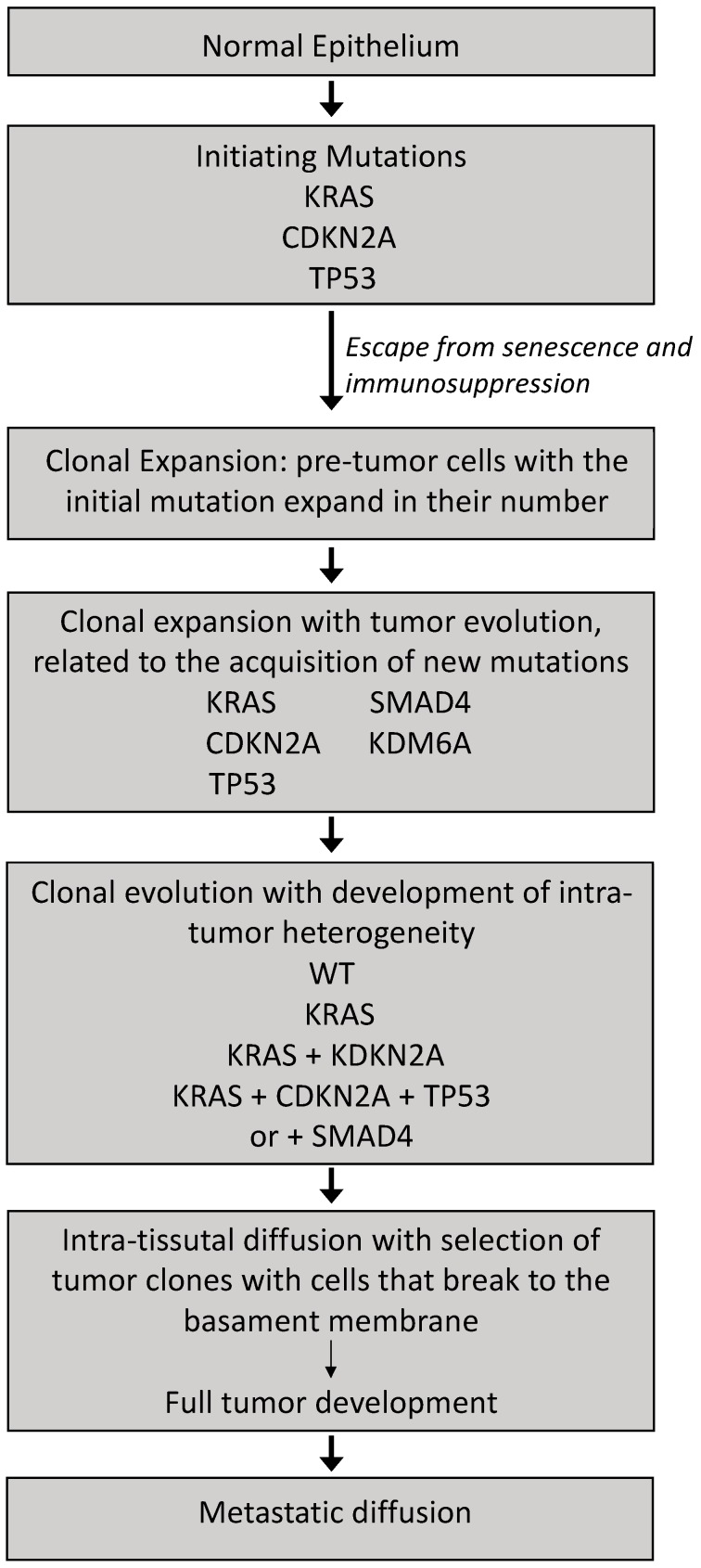
Various stages of pancreatic cancer evolution. The various stages of pancreatic cancer evolution are outlined, together with the main genetic alterations occurring at these stages.

The analysis of the genetic events associated with these precursor lesions, as well as the study of pancreatic carcinogenesis and the characterization of the genetic alterations observed in pancreatic cancers allowed proposing the model of pancreatic cancer evolution outlined in Figure 2. Basically, this model implies that PDAC development is dictated by the progressive acquisition of genetic alterations (*KRAS*, followed by *CDKN2A*, then *TP53* and *SMAD4*), with a gradual evolutionary trajectory of cancer progression because each alteration is acquired independently.

**Figure 3:**
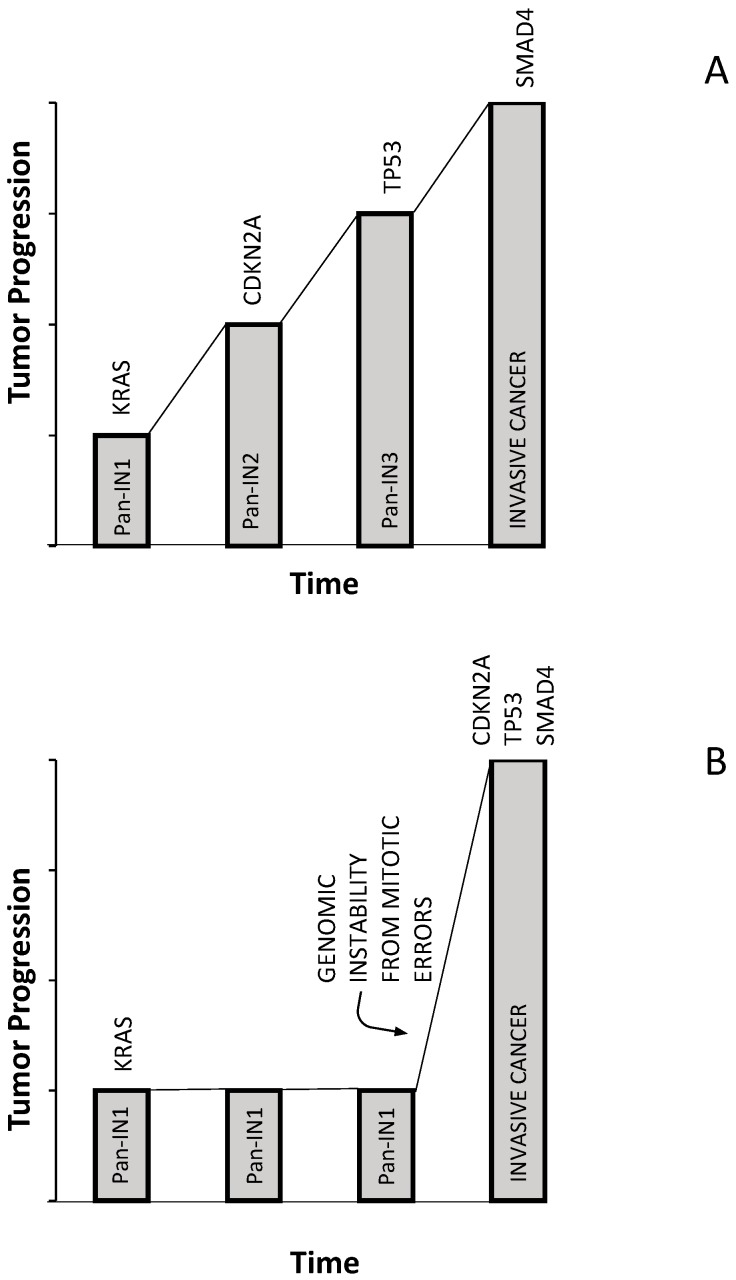
Two different models of PDAC development: (**A**) progressive accumulation of mutational events at various stages of tumor development; (**B**) after an initial transformation event, a unique chromothripsis event determines the rapid acquisition of additional mutations and copy number alterations. There is evidence that about 40% of PDACs develop following the A model and in about 60% of cases following the B model.

A recent study based on the analysis of copy number alterations occurring in PDACs provided evidence in favor of the PDAC evolution model based on genomic rearrangement patterns [34]. This important study showed that 45% of tumors displayed significant changes in copy number alterations, related to a phenomenon of polyploidization; of the polyploid tumors, 88% were tetraploid and 12% hexaploid [34]. Polyploid tumors have more *TP53* mutations. Using a sensitive algorithm (Chromothripsis Algorithm (ChromAL)), evidence was provided that copy number alterations are generated in 65% of PDACs through at least one chromothripsis event and not from localized events that accumulate over time [34]. Of all chromothripsis events, 11% occurred on chromosome 18, resulting in the loss of the tumor suppressor gene *SMAD4*; this event was accompanied by a gain in a region of chromosome 18 that harbors *GATA6* [34] (Figure 2). Eight percent of events occurred at the level of chromosome 12, leading to a focal amplification in the region of *KRAS* [34]. Polyploid tumors displayed chromothripsis events more frequently than diploid tumors and had a worse survival [34]. Some patients with multiple metastases were analyzed at the level of single metastasis, providing evidence for a model of PDAc development and progression implying that most mutations accumulate when these tumors are still diploid, and some of these mutations occur when these tumors are in a preneoplastic condition; this preneoplastic phase occurs during a prolonged time, preceding the malignant transformation, which requires copy number alterations and is accelerated by chromothripsis events [34] (Figure 3). This model implies also that the time of transition to malignant PDAC to metastatic disease is usually rapid, and this is consistent with the observation that 80% of PDAC patients present with advanced disease at diagnosis [34].

Whole genome sequencing and whole exome sequencing of PDACs have shown a mean mutation load of 1.8 and 1.1 mutation per megabase, respectively [35]. Only 5% of PDACs display the hypermutated phenotype [35]. The rare tumors with >12 somatic mutations/Mb display mismatch repair (MMR) deficiency and were 2/5 KRAS wild-type; the cause of MMR deficiency in these tumors was related to *MSH2* gene promoter deletion or mutation or *MLH1* gene promoter methylation [35]. The relatively more frequent tumors with a high tumor burden (4–12 mutations/Mb) frequently displayed homologous recombination repair deficiency [35]. The identification of these rare hypermutated PDACs is potentially important because the patients bearing these tumors are potential candidates for immunotherapy studies [35]. It is important to point out that the resolution of some genomic studies was confounded by the fact that PDAs are often highly desmoplastic, thus considerably lowering the tumor yield in tumor biopsies. Thus, some exome sequencing studies have reported relatively low mutation rates in PDA, lower than in other solid tumors; however, the low tumor cellularity seems to have contributed to this result. This interpretation is supported by the observation that Witkiewicz et al., using allele microdissection to enrich tumor epithelial cells prior to exome sequencing, found mutation rates three-times higher than those observed in studies not performing tumor cell enrichment by microdissection [36]. Thus, these authors observed in the exome 67 mutations per case [36]. The increased detection limit allowed identifying novel, recurrent mutations at a low frequency (<5%) in *IRF6*, *FLG*, *BCLAF1*, *AXIN1*, *GL13*, *PIK3CA* and *RBM10* genes [36].

Whole genome sequencing analyses have shown an average of 264 mutations per Mb in PDACs, thus indicating that these tumors carry thousands of mutations, most of which are in non-coding regions. Recently, Feigin and coworkers have developed a new computational system to analyze and classify non-coding mutational events in PDACs, providing evidence that most highly recurrent somatic non-coding mutations occur near genes in well-known PDAC-associated pathways, such as axon guidance, cell adhesion and Wnt signaling [37]. These observations suggest that non-coding mutations could drive PDAC progression by activating PDAC-specific pathways, cooperating with coding mutations [37].

The genetic diversity of pancreatic cancer was observed not only at the level of the somatic gene mutations, but also al the level of the transcription expression profile. According to the findings of this analysis, Collison and coworkers defined three subtypes of pancreas adenocarcinoma: classical, quasi-mesenchymal and exocrine-like. The classical sub-type had high expression of adhesion-associated and epithelial genes; the quasi-mesenchymal subtype had high expression of mesenchyme-associated genes; the exocrine-like subtype displayed high expression of tumor cell-derived digestive enzyme genes [20]. The survival of patients with the classical subtype following surgical resection and standard medical treatment was significantly better than that observed in individuals with the quasi-mesenchymal subtype; individuals with the exocrine-like subtype exhibited a survival rate intermediary between the two other subtypes [20].

Recent studies have stressed the importance of the stromal component in the prognostic stratification of PDAC and in the identification of tumor subtypes. PDAC fibrosis compromises drug delivery, limits accessibility of immune cells to the tumoral tissue and promotes disease aggression and chemoresistance. In a first study, Moffitt and coworkers have used a virtual microdissection technique to separate tumoral cells from the stromal cells and to analyze the gene expression pattern in these components: using this approach, they have identified a “normal” and an “activated” stromal subtypes from one side and a “classical” and “basal-like” subtype from the other side [38]. Patients whose samples belonged to the “activated stromal” subtype have a worse prognosis with a median survival time of 15 months and a one-year survival rate of 60%, compared to patients pertaining to the “normal stromal” subtype, with a median survival time of 24 months and a one-year survival rate of 82% [38]. Concerning the tumor-specific subtypes, patients with basal-like subtype had a worse median survival (one-year survival rate of 44% in comparison with a one-year survival rate of 70% for patients with tumors pertaining to the “classical type”) [38]. Basal-like and classical tumors were found in both the normal and activated stromal subtypes; however, patients with the classical subtype with the normal stromal subtype is a group of patients with good prognosis, while patients with tumors from the basal-like subtype with the activated stromal subtype have a very negative prognosis [38]. In a second study, Laklai and coworkers by using clinical specimens and various mouse experimental models identified a unique, highly rigid, matricellular-stromal tumor phenotype linked to a peculiar PDAC genotype resulting in deficient TGF-β signaling and elevated tumor cell contractility [39]. These observations suggest that genetically-induced cell tension represents a major determinant of the composition and mechanics of the periductal stroma in PDAC, by activating mechano signaling pathways related to β-integrins and YAP and inducing tumor aggression and epithelial-to-mesenchymal transition [39]. This stromal phenotype is largely reminiscent of the “activated stromal” subtype described by Muffitt.

Pancreatic carcinomas with acinar differentiation form a separate group of pancreatic cancers, including pancreatic acinar cell carcinoma (PACC), pancreatoblastoma and carcinomas with mixed differentiation. Two recent studies have analyzed the mutational profiles of this pancreatic cancer (PC) subtype. In a first study, Jiao and coworkers have observed a mean number of 199 somatic mutations per tumor [40]. Some genes mutated in PACs were also mutated in PACCc, but at a lower frequency, such as: *SMAD4* (26%), *TP53* (13%), *GNAS* (9%), *RNF43* (4%) and *MEN1* (4%) [40]. In addition, there is another set of genes not mutated in PACs, but frequently mutated in PACCs, such as *JAK1* (17%), BRAF (13%), RB1 (13%), *APC* (9%), *PTEN* (9%), *ARID1A* (9%), *MLL3* (9%) and *BAP1* (4%) [40]. A second study of genomic profiling of PACCs showed recurrent *BRAF* and *RAF1* rearrangements occurring in 23% of cases [35]. The most prevalent fusion was *SND1-BRAF* and resulted in activation of the MAPK pathway [41]. PACCs lacking *RAF* rearrangements frequently (45% of cases) display genomic alterations causing inactivation of DNA repair genes [41]. Furukawa and coworkers have performed the exome sequencing of 11 acinar cell carcinomas and identified recurrent mutations of BRCA2 and FAT genes: somatic or germline premature *BRCA2* mutations were observed in three of seven tumors, while *FAT1*, *FAT3* and *FAT4* somatic or germline mutations were observed in four of seven tumors [42]. According to these findings, it was concluded that PACC may commonly harbor somatic or germline loss-of-function mutations of *BRCA2* and fat genes [42]. Although the APC gene was mutated in only <10% of PAAC patients, APC loss of expression was observed in 48% of these tumors, gene silencing through epigenomic modifications being frequently involved in the repression of APC gene expression [43]. *TP53* mutations were observed in 13% of PACCs at the level of primary tumors; however, the frequency of *TP53* mutations was higher at the level of metastatic tumor specimens (31%) [44].

It was estimated that about 10% of pancreatic cancers have an inherited component, but the genetic basis for familial aggregation in these cases has not been identified. Analysis of the genetic alterations present in tumors has shown that both familial and sporadic pancreatic cancer share the same somatic molecular abnormalities [45]. A hereditary predisposition to develop pancreatic cancer manifests in three clinical settings: hereditary tumor predisposition syndromes such as hereditary breast ovarian cancer and Peutz-Jeghers syndrome; hereditary pancreatitis; familial pancreatic cancer (FPC). A recent study based on the screening of a large cohort of patients confirmed that FPC represents 9% of pancreatic cancer; the risk of malignancy in these families does not appear to be confined to the pancreas [46]. Patients with FPC have more precursor lesions, but for many, clinicopathologic factors and outcome are like those in patients with sporadic pancreatic cancer [46]. The exome sequencing of a large set of patients with familial pancreatic cancer demonstrated that inherited pancreatic cancer is highly heterogeneous: in these patients, germline mutations of *ATM*, *BRCA2*, *CDKN2A* and *PALB2* are observed, all elevating risk of developing pancreatic cancer [47]. Furthermore, deleterious variants in *BUB1B*, *CPA1*, *FANCC*, *FANCG* and *APC* are more frequent in patients with a familial history of pancreatic cancer [47]. Interestingly, a recent study based on the analysis of normal DNA from 854 patients with PDACs provided evidence that about 4% of these patients bear deleterious germline mutations [48]. Of 33 patients with deleterious germline mutations, 12 displayed deleterious germline *BRCA2* mutations, 10 *ATM* mutations, 3 *BRCA1* mutations, 2 *PALB2* mutations, 2 *MLH1* mutations and 1 each with a *CDKN2A* and *TP53* mutation [48]. Therefore, germline mutations in pancreatic cancer susceptibility genes are commonly identified in patients with pancreatic cancer without a significant family history of cancer. Other studies have shown that *BRCA2* causes 3–5% pancreatic cancers in population [49,50] and clinic-based cohorts [51], thus indicating that this gene is the most common high-penetrant cause of pancreatic cancer.

**Figure 4:**
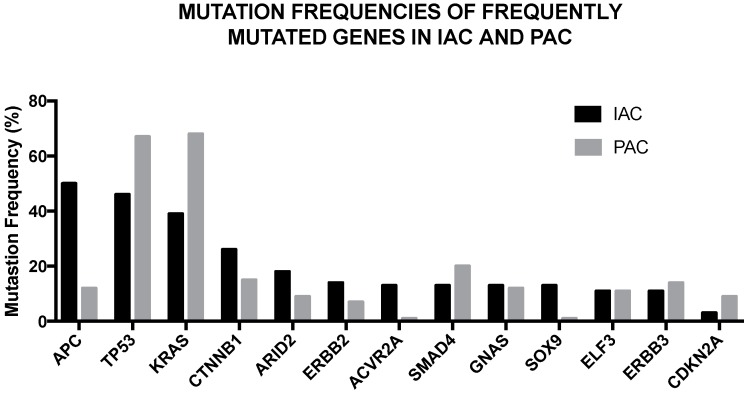
Frequency of the main genetic abnormalities observed in ampullary carcinomas, subdivided into intestinal-type (IAP) and pancreatobiliary-type (PAC).

Recent studies have characterized the genetic abnormalities observed in ampullary carcinomas, i.e., in the carcinomas originated at the level of the ampulla of Vater, an anatomical site where there are duodenal pancreatic and biliary epithelium merged to form the epithelium of the ampulla. Ampullary carcinomas can be separated into two histological subtypes, intestinal-type (IAC) and pancreatobiliary-type (PAC), with different pathogenic and clinical characteristics. Two recent studies have reported the results of a large integrative genomic analysis in two large sets of ampullary carcinomas [52,53], showing the spectrum of driver mutated genes observed in IACs and PACs (Figure 3). Interestingly, the mutational spectrum of IACs is more like that of colorectal cancer, while that of PDACs is more like pancreatic cancer [52,53]. Similarities in the mutated genes are observed in IACs compared to PDACs, but some remarkable differences have been observed between these two different tumors (Figure 4). This analysis showed also that alterations of the WNT pathway are more common in the IAC type, while RTK/RAS signaling and TP53/Rb signaling are more common in PAC [52,53].

Adenosquamous carcinoma of pancreas (ASCP), a tumor containing both squamous cell carcinoma and ductal adenocarcinoma components, is a rare malignancy that constitutes only about 1–4% of all exocrine malignancies. Due to the rarity of this condition, only limited genomic data have been generated. Recently, Fang and coworkers reported the whole-exome and whole-genome sequencing of 17 ASCP tumors, providing the largest available characterization of these tumors [54]. The tumor mutational burden was similar between ASCP and PDAC [54]. The most frequently mutated genes in ASCP were *KRAS*, *TP53* and *SMAD4*, with mutational frequencies like those observed in PDAC [54]; *TP53* gene was very frequently (88%) mutated in ASCP [54]. Copy number alterations are frequent and affect genes involved in cancer development and progression, such as *KRAS*, *CDKN2A*, *TP53*, *MYC*, *SMAD4* and *FHIT* [54]. Interestingly, multiple chromosome 3p regions in ASCP displayed gene copy number loss more frequently than in PDAc and determine tumor suppressor inactivation [54]. 3p loss was described as a frequent alteration of pancreatic endocrine neoplasia. Interestingly, in a few cases, the squamous component was isolated from the adenoma carcinoma component: both of these components displayed the same mutational spectrum, suggesting that the origin of these two histological components is the same [54]. Interestingly, Liu and coworkers have reported the very frequent mutations of the gene *UPF1* (Up-frameshift 1) in ASCP, observed in >80% of patients [55]. UPF1 is required for mRNA discrimination during nonsense-mediated decay (NMD), allowing one to discriminate faulty RNAs from normal RNAs. The mutated UPF1 observed in ASCP is defective in its function, and abnormal RNA transcripts (i.e., *TP53* gene transcripts) were observed in these tumors [55].

## 3. Genetic Abnormalities Involved in PDAC Metastasis

The genetic basis for metastasis in PDAC was recently explored. As above outlined, the most frequent initiating event in PDAC development is represented by an activating mutation of *KRAS* in acinar or ductal cells, sufficient to initiate a pre-malignant lesion. The subsequent step of tumor progression is triggered by inactivating mutations of tumor suppressor genes, such as *TP53*. However, no recurrent-specific, metastasis-specific gene mutations have been identified. As above outlined, the analysis of matched primary and metastatic PDAC samples provided evidence that the main driver mutations are maintained in the metastases compared to the primary tumors.

The study of some animal models allowed defining the possible genetic determinants of the metastatic behavior of pancreatic cancer cells. In this context, mice engineered with expression of mutant *KRAS* and *TP53* (KPC mice) at the level of pancreatic tissue develop autochthonous tumors of the pancreas that mimic human PDACs for their histological features and metabolic potential. Interestingly, a recent study introduced in these mice a floxed *SMAD4* allele, allowing conditional deletion of this tumor suppressor gene: the primary PDAs developed in these mice (KPDC mice) progressed locally more rapidly, but generated less metastases [56]. This reduced metastatic behavior was related to the reduced levels of the transcription factor RUNX3, overexpressed in KPC mice; the levels of RUNX3 define the different metastatic potential of these two disease presentations. In line with these observations, a relationship was observed between RUNX3 level in tumor epithelia and patient survival. Particularly, it was shown that the mutational status of *TP53* and the gene dosage of *SMAD4* cooperate to define RUNX3 levels: TP53 mild-type induces RUNX3 degradation, while inactivating TP53 point mutations stabilize it; *SMAD* gene dosage regulates RUNX3 levels in a biphasic manner, facilitating RUNX3 expression when both alleles are intact, inhibiting it with loss of one *SMAD4* allele and recovering RUNX3 expression when both *SMAD4* alleles are lost [56].

Seemingly, all patients possess cells with metastatic potential in their primary tumors at diagnosis [57]. The probability to metastasize varies as a function of tumor size, being 28% at 1 cm and 94% at 3 cm [45]. Although primary and metastatic tumors are very similar from a genetic point of view their capacity of tissutal proliferation is considerably variable since it is greatly influenced by tumor microenvironment [57]. Using the data of exome sequencing studies, an evaluation of the timing of progression of PDAC from precursor lesions to metastatic disease was attempted. In this context, Yachida et al. have provided an estimation of 11.7 ± 3.1 years for initial stages of PDAC development for the formation of PanIN and development of the first malignant clone (stage T1) and 6.8 ± 3.4 years for the development of subclones with malignant potential (stage T2) and 2.7 ± 1.2 years for the development of tumor metastases (stage T3) [15,58]. Similarly, Podolskiy and coworkers estimated a time of about 7–14 years for initial tumor development [59].

Some genes regulating tumor microenvironment and particularly the epithelial-to-mesenchymal transition could play a relevant role in PDAC metastasis. Thus, the EMT-transcription factor Zeb1 controls the colonization capacity and the phenotypic plasticity of tumor cells and promotes PDAC metastasis [48]. Depletion of Zeb1 suppresses stemness, colonization capacity and phenotypic/metabolic plasticity of PDAC cells [60]. In line with these observations, high Zeb1 levels in PDAC are associated with a poor prognosis [61].

An important role in PDAC metastasis could be related to alterations in epigenetic pathways. As mentioned above, whole-genome sequencing studies have provided evidence that various mutations of chromatin modifiers are recurrent events in PDACs and contribute to an alteration of the epigenetic mechanisms, playing a relevant role in tumor progression. A recent study provided evidence that disruption of large heterochromatin domains characterizes the metastatic transition in PDAC, because of aberrant oxidative pentose phosphate metabolism [62].

Roe and coworkers have used a peculiar approach (organoid culture system) to identify genes whose alterations are involved in metastatic transition [51]. Using this experimental strategy, they revealed that the metastatic transition is accompanied by massive and recurrent alterations in enhancer activations in enhancer activity [63]. The transcription factor FOXA1 was identified as a master driver of enhancer activation in metastatic PDACs, a mechanism that renders these cells more invasive, less anchorage dependent for growth in vitro and more prone to metastasis in vivo [63]. The reprogramming of enhancer activity operated by FOXA1 determines the activation of a transcriptional program of embryonic foregut endoderm [63].

Recent studies have analyzed the extent of the heterogeneity of known driver mutations in naturally occurring metastases. Makohon-Moore and coworkers have analyzed by whole genome sequencing the 26 metastases from four patients with PDAC: identical mutations in known driver genes were present in every metastatic lesion for each patient studied; only passenger metastatic mutations are responsible for all intratumoral heterogeneity [64]. The uniformity of driver mutations at the level of metastases in the same patient has fundamental implications for the possible success of targeted therapies [64].

It is evident that the preferential metastasis of PDACs to some peculiar tissues is dictated not only by the intrinsic properties of pancreatic cancer cells, but also by the anatomical position of pancreas and its circulatory system and by the peculiar microenvironmental properties of tissues more frequently colonized by metastatic PDACs. In these studies, peculiar emphasis was given to the liver, the most frequent site of PDAC metastases. Pancreas tumor cells secrete tissue inhibitor of metalloproteinase-1, which travels via the circulation to the liver, where it activates CD63^+^ hepatic stellate cells to produce stromal derived factor 1 (SDF-1) and, through this mechanism, to create a premetastatic hepatic niche [65]. The hepatic growth of metastatic PDAC cells is favored by activation of CD68^+^-associated macrophages, which secrete granulin, in turn acting on hepatic stellate cells, inducing their differentiation to myofibroblasts, able to release the periostin necessary to sustain the growth of metastatic PDAC cells [66]. Furthermore, the expression of CXCR2 by innate myeloid cells in the primary tumor microenvironment is fundamental for PDAC metastasis, as supported by two observations: either CXCR2 inhibition or depletion of neutrophils/myeloid-derived suppressor cells reduces metastases [67]. The CXCR2 is induced by mutated *KRAS* in pancreatic cancer cells and is required for autocrine growth of tumor cells [68] and for inhibition of oncogene-induced senescence [69].

## 4. Genetic Abnormalities of Pancreatic Intraductal Tubulopapillary

In the pancreas, in addition to IPMNs, a new type of intraductal neoplasms was recently identified and characterized at cellular and molecular level: intraductal tubulopapillary neoplasm (IPTN). IPTNs are rare intraductal neoplasms, characterized by absent or rare mucin production, and are characterized at the histological level by a tubular architecture, with only limited and sparse formation of papillary elements. These tumors are associated in about 40% of cases with invasive carcinoma, but their clinical behavior is less aggressive than that observed in classical PDACs.

As mentioned above, IPMNs are characterized by frequent *KRAS* mutations, *TP53* and *CDKN2A* mutations in cases with high-degree dysplasia, GNAs mutations in cases with high-degree dysplasia, *GNAS* mutations in 50% of cases (particularly in the intestinal subtype) and frequent RNF43 gene mutations. Only a few studies have characterized the molecular abnormalities of IPTN, providing evidence that they have a different mutational landscape, compared to IPMNs. Thus, the initial studies based on the analysis of only very limited number of IPTN tumors showed, at variance with IPMN tumors, a low frequency of *KRAS* (0–10%), *GNAS* (0–25%), TP53 (= −23%), *SMAD4* (0–10%) and *RNF43* (0–10%) mutations; more frequent were the CDKN2A alterations (54%) reviewed in [70]. In a recent study, Basturk and coworkers reported the analysis of the molecular abnormalities occurring in 22 IPTN tumors, showing that: most of the previously-reported IPMN genetic abnormalities were absent; loss of *CDKN2A* was observed in 25% of cases; *MAPK* genes were not frequently altered; chromatin remodeling genes (such as *MLL1*, *MLL2*, *MLL3*, *BAP1*) were altered in 32% of cases; PI3K pathway genes were altered in 27% of cases; finally, 18% of tumors displayed *FGFR2* fusions [71]. According to these data, it was concluded that IPTN is a distinct clinicopathologic entity and is genetically distinct from IPMN and PDAC [71].

In conclusion, PDAC is associated with different precursor lesions that impact the biology, the histotype, response to therapy and prognosis. The two main precursor lesions of PDAC are PanIN and IPMN. The actual evidence indicates that PanINs are the precursor lesions of classical PDAC and are responsible for the development of about 90% of these tumors [72]. IPMNs are responsible for the development of the remaining 10% of PDACs, and it was suggested that intestinal type IPMNs mainly originate colloid PDACs (a rare variant of PDAC), while intestinal type IPMNs mainly originate tubular PDACs, tumors morphologically identical to classical PDACs [72]. The analysis of molecular markers allows one to clearly distinguish invasive carcinoma derived from IPMNs (observed in some patients), from concomitant PDAC occurring together with IPMNs (observed in other patients) [73]. In contrast, IPTN lesions cannot be considered as precursors of PDAC, but originate pancreatic tumors with a different landscape of genetic abnormalities.

## 5. Animal Models of Pancreatic Cancer Development

Several studies have tried to identify and define somatic genetic abnormalities that can cooperate with driver mutations to induce the development of pancreatic cancer. To identify cooperating mutations, animal models of pancreatic cancer have been developed. These animal models are based on the expression of oncogenic KRAS in the pancreas either alone or in combination with inactivating alleles of homologs of known human pancreatic cancer drivers, including TP53 and SMAD4.

Some studies have explored the possible oncogenic cooperation between *KRAS* mutations and *SMAD4* loss. SMAD4 is an intracellular common mediator of the TGF-β superfamily signaling pathways. *SMAD4* loss in PDAC causes various phenotypic changes, including increased E-cadherin and CD133 expression, increased MAPK activation and increased chemoresistance [74]. SMAD4 loss is frequently observed in pancreatic cancer (more than 50%), but it is an event occurring at late stages of cancer development since it is rarely observed in pre-invasive pancreatic neoplasia. The analysis of the constitutively active allele of *KRAS* (*KRAS*^G12D^) in the ductal pancreatic epithelium elicited all the spectrum of the pre-invasive neoplastic lesions. Therefore, it seemed particularly interesting to evaluate a possible cooperation between *KRAS* mutation and *SMAD4* loss in favoring the progression of pancreatic cancer [75]. The results of these studies showed that the loss of *SMAD4* in conjunction with *KRAS*^G12D^ stimulated the progression of pancreatic cancer development through various mechanisms, including accelerated fibrosis, enhanced acinar cell loss and accelerated development of various pre-invasive neoplastic lesions [75].

However, it must be pointed out that the role of TGF-β signaling in PDAC development is complex. In fact, some studies have shown that conditional loss of *SMAD4* or *TGFBR2* in Pdx1-Cre/LSL-KRas mice develop advanced aggressive pancreatic cancer [76]. In contrast, systematic deficiency of *TGFBR1* in elastase-*KRAS*^GRD^ (EL-KRAS) mice induced a marked reduction in the tumoral phenotype [77]. These diametrically opposed findings can be explained considering the differential effect of TGF-β signaling at the level of tumor cells and of the tumor microenvironment: in pancreas cells, TGF-β deficiency favors tumor development and is associated with tumor progression; in contrast, TGF-β deficiency at the level of microenvironment protects against tumor development by promoting fibrosis and immune invasion [78].

On the other hand, other studies have shown that either *p16* or *p53* loss, in conjunction with constitutive *KRAS*, promoted pancreatic cancer progression, thus indicating that both the retinoblastoma and the *p53* pathway are involved in the suppression of pancreatic cancer development [79].

All these models showed the progressive development of neoplastic lesions, corresponding to murine intraepithelial neoplasia, that in some cases become invasive or progress to adenocarcinoma. In spite that these models have considerably contributed to the progress of our understanding of pancreas cancer biology, however, they have not allowed the identification of cooperating mutations required for neoplastic disease progression. To identify these mutations, another strategy was used, consisting of performing an insertional mutagenesis screen using the inducible Sleeping Beauty (SB) transposon system in combination with an oncogenic KRAS pancreatic cancer model. SB favors the development and progression of pancreatic neoplasia compared to KRAS alone and allows one to explore all the stages of tumor development. Using this approach, 543 candidate cancer genes have been detected, and 75 of them, including *MLL3* and *PTK2*, have known mutations in human pancreatic cancer patients [80]. It is important to underline that 10% of these genes are involved in chromatin remodeling, including *ARID4B* and *NSD3*; finally, 20 of the identified genes are associated with poor survival [80]. The same approach was used by other investigators reporting the identification of the X-linked deubiquitinase *USP9X* as the gene most frequently mutated in KRAS mice submitted to insertional mutagenesis: loss of *USP9X* gene enhances transformation and promotes tumor progression. In pancreatic cancer patients, low USP9X levels were associated with metastatic burden in advanced disease and with poor survival [81]. Evidence was provided that USP9X protein levels are low in most of pancreatic cancer cell lines: following treatment of the cells with chromatin modulating agents, USP9X levels are significantly increased. Finally, in disease animal models, conditional deletion of USP9X cooperates with oncogenic KRAS to induce pancreatic cancer development [81].

Another gene cooperating with mutated *KRAS* to induce pancreatic carcinogenesis is cyclin-dependent kinase 5 (CDK5). Eggers and coworkers have recently reported that CDK5 and its co-activators p35 and p39 are significantly overexpressed in more than 90%, 94% and 75%, respectively, compared with normal pancreas; the molecular mechanisms responsible for this overexpression are in part related to gene amplification, the genes encoding CDK5, p35 and p39 being amplified in 67% of pancreatic cancers [82]. Importantly, CDK5 is activated by induction of the KRAS signaling, and oncogenic KRAS and activated CDK5 cooperate to induce pancreatic tumorigenesis [82].

Other studies have addressed the important question of the cooperation between two genetic driver mutations in inducing pancreas tumorigenesis. Morton and coworkers have explored the cooperation between *KRAS* mutants and *TP53* inactivating mutations to induce pancreas cancer formation [83]. In this study, these authors showed that KRAS^G12D^, despite its role as an oncoprotein, induces a senescence program within pancreas cells: thus, most of the mouse pancreas cells transformed with KRas^G12D^ die via senescence, and the surviving cells form PanIN lesions that rarely progress to pancreatic cancer [83]. Loss of *TP53* or inactivating mutations of TP53 impede the senescence of KRAS^G12D^ and favor the malignant transformation of these cells [83]. An additional effect elicited by TP53 loss in these models consisted of the promotion of tumor metastases [83]. Therefore, these studies suggest two critical functions of TP53 in pancreatic cancerogenesis: escape from KRas^G12D^-induced senescence and promotion of metastasis [83]. The pancreatic cancer model based on the double KRAS^G12D^ and *TP53* mutation was used to explore the sensitivity of pancreatic cancer cells to new drugs. Using this mouse model, it was possible to demonstrate that minnelide, a triptolide analog, was highly effective at reducing pancreatic tumor growth and spread [84].

The exploration of KRAS-driven PDAC helped to better define the physio-pathological role of TP53. As mentioned above, wild-type TP53 acts as a potent tumor suppressor of pancreatic cancer development through a mechanism involving the protein tyrosine phosphatase called PTPN14, which bridges TP53 with another tumor suppressor network, the Hippo pathway [85]. These studies were based on the use of a p53 double-mutant acting as a “super rumor suppressor”; this super-suppressor hyper activates PTPN14, which, in turn, negatively regulates YAP protein, inducing its cytoplasmic sequestration and consequent degradation [85]. In the presence of a mutant TP53, this tumor suppressive effect is lost, and pancreatic tumorigenesis is promoted.

In addition to the above-mentioned mouse models, other models of KRas mutant/TP53 mutants were developed. In this context, particularly interesting were two pancreatic cancer models developed by Collins and coworkers [86]. These authors have reported two mouse models of pancreatic tumorigenesis: mouse transgenic for inducible KRas^G12D^, allowing the selective pancreas-specific expression of KRas^G12D^ with or without inactivation of one allele encoding the tumor suppressor TP53 [86]. Using these two cancer models and exploiting the inducibility of the Kras^G12D^ in these models, it was possible to evaluate the role of this oncogene at early and later stages of tumor progression. The results of these studies clearly showed that oncogenic KRAS is required for both the initiation and the maintenance of pancreatic cancer in mice [86]. Furthermore, during all the stages of tumor development, KRAS^G12D^ upregulated Hedgehog signaling, inflammatory pathways and several pathways known to be involved in the control of paracrine interaction between epithelial cells and the microenvironment, promoting through this mechanism the induction and the maintenance of a fibro-inflammatory stroma promoting tumor development [86].

Lee and coworkers have recently analyzed in detail the effect of HH signaling on pancreatic cancer progression in three different mouse models of KRas-induced pancreatic cancer. In humans, increased levels of Sonic Hedgehog are observed during pancreatic cancer. Genetic or pharmacologic inhibition of HH pathway activity in these mouse models accelerates progression of oncogenic KRAS-driven disease. Particularly, inhibition of HH signaling suppresses stromal desmoplasia, but caused accelerated growth of the neoplastic epithelium. In contrast, potentiation of HH signaling caused stromal hyperplasia and reduced epithelial neoplastic proliferation [87].

Other studies have explored the oncogenic interaction between activated mutant KRAS and the constitutively activated NF-κB pathway. In this context, previous studies have provided evidence that the NF-κB pathway is activated in about 70% of pancreatic carcinomas. Therefore, it seemed important to evaluate a possible cooperation between the RAS and NF-κB pathways in promoting pancreas carcinogenesis; furthermore, these studies explored also a possible involvement of NF-κB activation in mediating the oncogenic effects of KRAS^G12D^. The results of these studies showed that pancreas-targeted *IKK2* inactivation inhibited NF-κB activation and pancreatic neoplasia development in KRAS^G12D^ mice, thus suggesting an important role for NF-κB activation for KRAS-mediated pancreas tumorigenesis [88]. The explanation for this mechanistic link is related to the capacity of KRAS^G12D^ to activate AP-1, which in turn induces IL-1α, responsible for NF-κB activation [88]. In line with these observations, IL-1α was overexpressed in pancreatic cancer, its expression correlating with *KRAS* mutation, NF-κB activation and poor survival [88]. The synergistic effect of activated KRAS with NF-κB activation was further explored. Thus, Daniluk and coworkers have shown that inflammatory stimuli initiate a positive regulatory feedback loop involving NF-κB that further amplifies RAS activity of mutant *KRAS*. These inflammatory stimuli promoted cancer progression in mice expressing KRas^G12D^, which can be prevented by deletion of *IKK2* or inhibition of COX-2 [89]. These observations suggest that in the presence of a mutated *KRAS*, inflammatory stimuli promote pancreas cancerogenesis through an NF-κB-mediated positive feedback mechanism involving COX-2 that amplifies RAS activity to pathological levels [89].

An important role of inflammation in pancreatic tumor development is supported also by the analysis of mouse models based on the transformation of mouse acinar cells by mutant KRAS. The capacity of mutant KRAS to induce PanIN in mouse pancreatic acinar cells decreases with age and is completely abolished after P60 (postnatal day 60) [90]. Furthermore, KRAS^G12D^ expression in mature acinar cells did not induce tumor lesions even in combination with TP53 or CDKN2A deficiency [90]. However, these acinar cells yield pancreatic intraepithelial neoplasia and ductal adenocarcinomas if exposed to limited bouts of non-acute pancreatitis, providing they harbor KRAS oncogenes [90]. The inflammation contributes to tumor development and progression by abrogating the senescence barrier of low-grade PanINs [90]. Other studies have shown that the inflammatory-inducing effect on pancreatic tumorigenesis is mediated by the release of interleukin-13, which induces the conversion of inflammatory macrophages into alternatively activated macrophages, exhibiting tumor-promoting effects [91]. Treatment of mice expressing oncogenic *KRAS* with neutralizing antibody for IL-13 reduces tumor formation [91]. Pancreatic adult duct cells are still more resistant to oncogenic transformation by oncogenic KRAS expression. The transformation of these cells can be achieved only by the combination of KRAS^G12D^ overexpression and loss of TP53 and CDKN2A, only if CDKN2B expression is concomitantly inactivated [92].

Several recent studies have explored the mechanisms through which *KRAS* mutation may act as a potent driver of pancreatic carcinogenesis. Thus, Ying and coworkers, using a KRAS^G12D^-inducing mouse model, have performed a transcriptome and metabolomics analysis of KRAS-induced tumors, showing that KRAS^G12D^ plays a key role in the control of tumor metabolism through stimulation of glucose uptake and channeling of glucose intermediates into hexosamine biosynthesis and the pentose phosphate pathway [93]. In addition to these effects, KRas^G12D^ promotes also ribose biogenesis. However, since KRas^G12D^ triggers glycolysis intermediates into the nonoxidative pentose phosphate pathway, it decouples ribose biogenesis from NADP/NADPH-mediated redox control. These observations indicate that KRas^G12D^ promotes metabolic reprogramming [93]. Another recent study showed that oncogenic KRAS activation modifies the glutamine metabolism in pancreatic cancer cells. Thus, a non-canonical pathway of glutamine metabolism was found in pancreatic cancer cells: whereas normal cells use glutamate dehydrogenase (GLUD1) to catalyze the conversion of glutamate into α-ketoglutarate to sustain the tricarboxylic acid cycle for the production of energy required for cellular metabolism, in pancreatic cancer cells, glutamine-derived aspartate is transported into the cytoplasm, where it is converted into oxaloacetate by aspartate transaminase and, in turn, into malate and then pyruvate, increasing the NADPH/NADP^+^ ratio required to maintain the cellular redox state [94]. Because of this metabolic subversion, pancreatic cancer cells are exquisitely sensitive to glutamine deprivation, increasing their production of ROS, reduction of GSH and consequent inhibition of tumor growth/survival [94]. Other studies indicate that oncogenic KRAS acts as a modulator of redox response. In fact, it was shown that oncogenic RAS expression in pancreatic cancer cells induces NRF2 expression; NRF2 controls cell response to oxidative stress by transcriptional upregulation of antioxidant-response-element bearing genes [95]. NRF2 expression is necessary to maintain the proliferation of PDAC cells [95]. This transcriptional factor acts by regulating mRNA translation; loss of NRF2 led to defects in autocrine EGFR signaling and oxidation of specific translational regulatory proteins, resulting in impaired cap-dependent and cap-independent mRNA translation in PDAC cells [96]. Other abnormalities of oxido-reductases could play a relevant role in KRAS-mediated pancreatic tumorigenesis. Particularly, the study of the model of KRAS and p16-induced mouse pancreatic tumorigenesis provided evidence of the pathogenic role of NOX4 [97]. NOX enzymes are a family of NDPH oxidases that, together with their small membrane-bound catalytic subunit (p22^phox^), mediate the oxidation of NADPH to NADP^+^ or NADH to NAD^+^, which in turn leads to the formation of superoxide and other ROS. Ju and coworkers have explored a murine model of PDAC induced by mutant KRAS and p16 loss, where NOX4 activation was observed; NOX4 elevated activity accelerates oxidation of NADH and supports increased glycolysis by generating NAD^+^, thus promoting PDAC growth [97]. NOX4 was induced through p16-Rb-regulated E2F, and p22^phox^ was induced by KRAS^G12V^-activated NF-κB [97].

A set of studies was focused to evaluate the role of NUPR1, a basic helix-loop-helix molecule, in the oncogenic effects mediated by KRAS at the level of the pancreatic tissue. NUPR1 expression is strongly upmodulated by acute pancreatitis, and its levels are increased in pancreatic adenocarcinomas. NUPR1 plays a fundamental role in pancreatic tumorigenesis as shown by the observation that the oncogenic form of KRAS^G12D^ was unable to promote PanINs in the absence of NUPR1 [98]. In this study, it was shown also that NUPR1 protected pancreatic cancer cells from apoptosis through a pathway dependent on transcription factor Relb and immediate early response 3 (IER3) [98]. Using the same mouse model, it was possible also to demonstrate that NUPR1 cooperates with oncogenic KRAS^G12D^ to induce PanIN formation by modulating the expression of gene networks that are involved in the regulation of senescence [99]. Pancreatic cancers developed in KRAS-induced carcinomas and NUPR1-deficient mice exhibited a higher expression of stemness markers (ALDH1, SOX2, Oct-4) compared to tumors originated in NUPR1-WT mice [100]. A more recent study has classified the mechanism through which IER3, the gene whose expression is induced by NUPR1, contributes to NUPR1 cooperation in KRAS-induced oncogenesis. In fact, it was shown that IER3 supports KRAS^G12D^-associated oncogenesis in the pancreas by sustaining ERK1/2 phosphorylation through phosphatase PP2A inhibition [101].

The study of KRAS-dependent models of pancreatic tumorigenesis was of fundamental importance for the understanding of the cell of origin of pancreatic cancers and for the analysis of the whole process of pancreatic tumor development. As discussed above in detail, oncogenic *KRAS* mutations are fundamental drivers in pancreatic carcinogenesis. *KRAS* mutations represent also the earliest detectable mutations found in preneoplastic lesions [102]. In fact, Kanda and coworkers have studied many PanIN lesions at various stages and have observed that about 96.5% of PanINs harbored *KRAS* mutations [102]. In the earliest PanIN lesions, *KRAS* mutations are present in only a fraction of cells comprising the preneoplastic lesion [102]. In contrast, p16/CDKN2A (11.5% of cases), *GNAS* and *BRAF* mutations have been observed only in a minority of these PanINs [102]. These findings were confirmed through the analysis of PanIN lesions in a group of patients with a family history of pancreatic cancer: 95% of these lesions harbored *KRAS* mutations [25,103].

In the KRAS^G12D^-driven model of murine pancreatic tumorigenesis, cancer formation is preceded by PanIN; in these mice, PanIN formation is associated with or is preceded by acinar-to-ductal metaplasia, characterized at the phenotypic level by replacement of acinar cells with cellular elements exhibiting the expression of CK19 and Sox9, a transcriptional determinant of ductal cell fate [104]. According to these findings, one can conclude that *KRAS* mutations induce acinar-ductal metaplasia (ADM), PanINs and, finally, ductal pancreatic cancer. However, based on these findings, it is impossible to distinguish between two different cellular mechanisms: (a) ADM and PanINs are generated by the expansion of ductal cells, with consequent progressive replacement of acinar cells by these cells; (b) ADM and PanINs are originated from the reprogramming of acinar cells into ductal-like cells. The studies carried out at the level of KRAS-induced murine pancreatic cancers have in part clarified this issue. In this context, the initial studies of KRAS-mediated pancreatic tumorigenesis have used Pdx1^Cre^ or Ptf1a^Cre^ alleles to activate KRAS at the level of embryonic pancreatic progenitors, but did not allow distinguishing the pancreatic cell lineage, acinar or ductal, involved in this tumorigenic process. The development of new Cre driver lines allowed addressing the problem of the adult pancreatic lineage involved in pancreatic tumorigenesis; in some of these studies, either the nestin-Cre driver was used to activate KRAS in the exocrine pancreas progenitors and their acinar cell progeny [105] or other Cre drivers to activate KRAS in adult acinar cells [106].

The initial stages of KRAS-induced pancreatic oncogenesis are accelerated and potentiated by an inflammatory microenvironment. KRAS itself seems responsible for the induction of an inflammatory response at the level of the pancreatic tissue. In fact, it was shown that KRAS induces expression of IL-17 receptors on PanIN epithelial cells and stimulates the production of IL-17 by pancreatic IL-17 producing T helper cells, thus determining a paracrine mechanism of inflammatory response [107]. IL-17 plays an active role in chronic inflammation, and its enforced expression strongly accelerates PanIN initiation and progression, while inhibition of IL-17 signaling prevents PanIN formation [107].

Other models of pancreatic tumorigenesis have suggested an origin of murine pancreatic cancer from centroacinar cells. The pancreas-specific knockout of PTEN generated mice exhibiting the progressive replacement of their acinar pancreas with highly proliferative centroacinar cells, with properties of progenitor cells and generating a ductal-like population; a part of these mice develops ductal pancreatic malignancy [108]. According to these observations, it was suggested that centroacinar cells may represent the cells of origin of pancreatic cancer in mice [108]. It is important to note that the centroacinar cells remain an enigmatic cell type in the pancreas, and their exact cell lineage remains uncertain, while their capacity to act as pancreatic progenitors is evident [109].

On the other hand, studies based on the use of CK-19 promoter-based alleles, allowing activation of oncogenic KRAS in ductal cells and not in acinar cells, generated PanINs with low efficiency, thus suggesting that ductal cells (and particularly those pertaining to the large ducts) are not the cells of origin of pancreatic neoplasia [110].

The problem of the cellular origin of KRAS-induced murine pancreatic carcinoma was recently reassessed using a peculiar approach consisting of the induction of a KRAS mutation in pancreatic cells and then in labeling and tracing of the three main pancreatic cell populations: acinar, centroacinar and ductal [111]. The results of these studies showed that ductal and centroacinar cells are refractory to oncogenic transformation by oncogenic KRAS, whereas acinar cells are transformed by KRAS^G12D^, generating PanINs lesions with ductal features [98]. Using loss- and gain-of-function approaches, the transcription factor Sox9, a ductal fate determinant, was identified as a critical mediator of KRAS-mediated ductal reprogramming of acinar cells [111]. In line with these observations, the concomitant enforced expression of Sox9, together with KRAS^G12D^, accelerates the formation of PanINs [111].

As mentioned above, genetic profiling studies of resected human specimens have identified alterations in signaling pathways involving KRAS and GNAS signaling as early events in the pathogenesis of IPMNs. Given this background, it seemed particularly interesting to evaluate the existence of a possible cooperation between oncogenic KRAS (G12D) and oncogenic GNAS (R201H). Transgenic mice expressing the two oncogene proteins developed cystic tumors, consisting of markedly dilated ducts lined with papillary dysplastic epithelia in the pancreas, closely mimicking human IPMNs [112]. These observations clearly support a role of KRAS and GNAS mutations in the cooperative promotion of murine pancreatic tumorigenesis, closely recapitulating IPMN [112].

Additional evidence suggests a role for Sox9 in acinar to ductal metaplasia. In fact, Reichert et al. have performed an interesting study, starting from the hypothesis that a common transcriptional program could control embryonic ductal development, acinar-to-ductal metaplasia and PanIN formation since all these processes involve a ductal phenotype [113]. Using various techniques, these authors identified the homeodomain transcriptional factor PRRX1 [113]. The PRRX1 transcription factor generates two isoforms, PRRX1a and PRRX1b, which are both upmodulated in pancreatitis and neoplastic pancreatic transformation, including KRas^G12D^-induced PanINs [113]. Interestingly, the PRRX1b isoform, which is clearly upmodulated during acinar-to-ductal metaplasia, binds to the Sox9 promoter and upmodulates Sox9 expression [113]. This study, together with the previous study, indicates that the PRRX1-Sox9 axis acts as a positive modulator of the acinar-to-ductal metaplasia [113].

A very recent study provided a more careful definition of the effects of oncogenic KRas^G12D^ at the level of the differentiation process of pancreatic progenitor cells and implied also a possible effect at the stem cell level. To this end, Ischenko and coworkers have isolated pancreatic cells from KRASG12dp53-mice and observed that these cells largely expressed a stem cell phenotype (positivity for CD44, EpCAM and CD24): these cells were screened for Sca1 expression, showing that about 80% of the cells are Sca1-positive, are tumorigenic and, when transplanted into mice, generated tumors of the ductal phenotype/morphology; the remaining 20% are Sca1-negative, are tumorigenic and, when transplanted, generated sarcomatoid undifferentiated tumors [114]. The Sca1-negative population exhibited an increased tendency to form tumor spheres and to generate metastasis and expressed c-Myc; interestingly, c-Myc expression in Sca1-positive cells induced the generation of Sca1-negative cells with increased tumor sphere-forming capacity and increased metastatic potential [101]. These observations indicate that c-Myc plays an essential role in the control of self-renewal and lineage commitment with metastatic pancreas cancer cells. Other studies carried out by the same authors were prompted by the observation that a restricted population of adult pancreatic cells expressing the pancreatic and duodenal homeobox 1 (PDX1) is particularly sensitive to transformation by oncogenic KRAS. PDX1 is a transcription factor expressed in early pancreatic precursor cells and in insulin-producing pancreatic cells. Using the Lox-Stop-Lox-KRasG12D genetic mouse model of pancreatic carcinogenesis, a population of KRAS-expressing PDX1^+^ cells was isolated: these cells have a stem-like phenotype (EpCAM^+^CD24^+^CD44^+^CD133^−^Sca1^−^) and have the capacity to metastasize [115]. These cells are tumorigenic only when they remain in their undifferentiated state [115]. These observations suggest that adult pancreas harbors a dormant cell population that is capable of initiating tumor growth when appropriately stimulated by an oncogenic stimulation [115]. The analysis of this model suggested also that mitogen-activated kinase and c-MYC stabilization are the main driving forces for the development of an aggressive metastatic pancreatic cancer [115].

Recent studies have provided evidence that the role of PDX1 in pancreatic cancerogenesis is complex. This homeobox transcription factor is expressed in the pancreatic anlage and is required for differentiation of all pancreatic cell lineages; in pancreatic tissue, PDX1 expression is high in β-cells, where its expression is required for insulin expression, and low in exocrine cells. In spite of the low expression of PDX1 in acinar cells, this homeobox is required in maintaining acinar cell differentiation and represents a factor contributing to the mechanisms of resistance of these cells to neoplastic transformation [116]. Following malignant transformation, the role of PDX1 changes from tumor-suppressive to rumor-promoting [116]. In PDACs, PDX1 expression is very high in the ADEX, pancreatic progenitor and immunogenic subtypes; in contrast, the squamous subtype displays only low levels of PDX1 expression, due to hypermethylation of the PDX1 gene promoter [116]. Low PDX1 expression in PDACs was associated with poor prognosis. The low expression of PDX1 in pancreatic cancer cells favors the epithelial-to-mesenchymal transition and represents a mechanism contributing to the progression in malignancy [116].

Other studies have tried to define the molecular signaling pathways that are instrumental to induce the KRAS^G12D^-mediated induction of acinar-to-ductal metaplasia. Using a three-dimensional culture system, evidence was provided that KRas^G12D^ expression at the level of acinar cells rapidly induced acinar-to-ductal metaplasia with silencing of acinar genes and induction of duct genes [117]. Among the various signaling pathways activated by oncogenic KRAS, the Raf/MEK/ERK pathway seems to play a relevant role in ADM induction [117]. Interestingly, the enforced expression of the acinar-specific transcription factor Mist1, which is a critical controller of acinar cell organization, resulted in an inhibition of KRAS^G12D^-mediated ADM [117].

Oncogenic KRAS activates many signaling pathways, and a recent study indicates that the PI3K/AKT pathway could play a relevant role in the oncogenic activity of KRAS and in its metaplastic-inducing activity. Thus, Eser and coworkers have provided evidence that PI3K, as well as 3-phosphoinositide-dependent kinase 1 (PDK1) are activated in tumor pancreatic neoplasia (including preneoplastic lesions), as well as in KRAS-driven murine pancreatic cancer [118]. Importantly, blockade of PI3K or PDK1 activity elicited a marked inhibition of KRAS-dependent metaplasia and tumorigenesis, thus suggesting also that the KRAS→PI3K→PDK1 pathway may represent a potentially important target in pancreatic cancer therapy [118].

The exploration of an inducible KRAS^G12D^-driven mouse model of pancreatic ductal adenocarcinoma provided evidence about some molecular pathways responsible for KRAS^G12D^ independent tumor recurrence [119]. In fact, Kapoor and coworkers have observed some tumors undergoing spontaneous relapse: these tumors are devoid of KRAS^G12D^ expression and show amplification and overexpression of the transcriptional coactivator Yap1 [119]. Functional studies have shown that Yap1, in cooperation with the transcription factor Tead2, drives KRAS^G12D^ independent tumor maintenance, through a molecular mechanism involving activation of cell cycle and DNA replication program [119]. Another recent study explored the capacity of PDAC cells to survive to a complete deletion of endogenous KRAS function [120]. The dependency of human PDAc cell lines from KRAs activity is variable, and it was possible to isolate PDAc cells able to survive in the absence of KRAS activity; these cells are particularly sensitive to PI3K inhibitors, thus offering a pharmacologically suitable strategy to subvert resistance to KRAS blockade [120].

In conclusion, the recent evidence emerging from the study of mouse models and lineage tracing experiments indicates that pancreas ductal adenocarcinoma develops in the centroacinar-acinar cell compartment through a process of acinar-to-ductal metaplasia or through the expansion of centroacinar cells accompanied by apoptosis of acinar cells. However, these results must be considered with great caution, for that concerns their possible extrapolation at the level of spontaneously occurring human pancreatic ductal adenocarcinoma. In fact, studies carried out in human lesions have suggested that ADM and PanIN are morphologically and genetically distinct [33,121,122]. In fact, importantly, only ADM lesions associated with PanIN lesions display KRAS mutations, while isolated ADM, without PanIN, do not display KRAS mutations [33,122]. Furthermore, all acinar cell foci located near PanIN failed to display KRAS mutations [33]. These findings were compatible with a model implying the origin of human pancreas cancer not from acinar cells [33]. At variance with these findings, the accurate analysis of the pancreas of patients with a strong family history of pancreatic cancer has shown a high incidence of PanINs and has suggested a role for these lesions in cancer development. In addition, in these patients, a high incidence of ADM was observed; some of these ADM areas were characterized by the presence of flat atypical lesions, which could represent regions of increased proliferation of cells with precursor potential. According to these findings, like those observed in the pancreas of KRas^G12D^ mice, it was suggested that at least in these patients, ADMs could represent the starting lesions, developing later into cancer [123].

Using models of pancreatic intraepithelial neoplasia, evidence was provided that the microtubule regulator DCLK1 (doublecortin and Ca^2+^/calmodulin-dependent kinase-like 1) is a marker of a distinct subpopulation of cells with stem cell properties [124]. These DCLK1^+^ cells displayed morphological and molecular features typical of gastrointestinal tuft cells [103]. Pharmacological treatment with gamma-secretase inhibitors greatly reduced the proportion of DCLK1^+^ cells at the level of PanIN [124].

As outlined above, the data obtained using genetically-engineered mouse models support the genetic PDAC progression model and have led to the hypothesis that both acinar cells and ductal cells have the potential to generate invasive PDAC via different precancerous developmental routes. However, human cell-based models of PDAC development are not available. A recent study reported the development of a pancreatic intraepithelial neoplasia model from primary human pancreas ductal cells. This model is based on gene delivery of KRAS, CDKN2A, TP53 and SMAD4 into primary human pancreatic ductal cells [125]. Immortalized ductal cells grow as epithelial monolayer spheres in three-dimensional cultures [125]. Following orthoptic transplantation into mouse adult pancreas, these cells generate in vivo structures displaying cellular and molecular features typical of PanIN. Therefore, this experimental system represents a unique tool to explore cellular and molecular mechanisms responsible for PanIN development from normal ductal cells [125]. However, these PanIN lesions do not progress to pancreatic cancer [125]. Importantly, intercellular or signaling interactions typically observed in PDAC development are recapitulated by PanIN cells after orthoptic transplantation, particularly concerning the acquisition of dysplastic features of cells present in PanIN and the fibroblastic reaction of surrounding stroma [125].

## 6. Epigenetic Abnormalities in Pancreatic Cancer

Besides some driver genes that are frequently mutated in pancreatic cancer, there are several oncogenes that do not carry mutations, but are deregulated in their expression and contribute to tumor maintenance/progression. A prototype of these oncogenes in pancreatic cancer is c-Myc for its overexpression observed in a part of pancreatic cancers, due, more frequently to enhanced transcriptional activity and, more rarely, to gene amplification; because of these events, the c-Myc core signaling is one of the pathways more frequently activated in pancreatic cancer [6]. To study the role of c-Myc overexpression at the level of the exocrine pancreatic lineage, Lin and coworkers have developed a mouse model implying a temporally- and spatially-controlled expression of c-Myc at the level of pancreatic progenitors [126]. Upregulation of c-Myc expression resulted in the rapid formation of ductal precursor lesions and of adenocarcinomas, metastasizing at the level of the liver [112]. The downregulation of c-Myc expression in these tumors induces cell death, thus indicating their dependency on c-Myc for their survival [126]. However, few tumor cells survived to c-Myc downregulation: these cells remained dormant, expressed stem cell markers and gave rise to tumor recurrence upon c-Myc re-expression [126].

EGFR is the example of another gene that is not mutated in pancreatic cancer, but whose expression is very frequently increased in this tumor. During the last two decades, several molecular agents able to target various signaling pathways have been explored to attempt the experimental treatment of pancreatic cancer patients. However, among the various agents tested, only the oral epidermal growth factor receptor (EGFR) tyrosine kinase inhibitor Erlotinib has shown a statistically significant, but clinically moderate, overall survival benefit when added to standard chemotherapy with gemcitabine in a large phase III clinical trial. EGFR was not mutated in pancreatic cancer, but is overexpressed in about 90% of these tumors [127]. Two recent studies have shown that EGFR induction and activation plays an essential role in KRAS-induced pancreatic tumorigenesis, and these are interesting findings for their potential therapeutic implications. These studies were stimulated from the observation that EGFR signaling is required for pancreatic metaplasia, a precursor of the pre-neoplastic lesions PanIN that can lead to pancreatic ductal carcinoma [128]. In line with these observations, elevated EGFR levels were observed in metaplastic lesions of pancreatitis patients and in pancreatic tumor lesions [127]. Importantly, in KRAS-induced murine tumor models, EGFR signaling is essential for KRAS oncogene-driven pancreatic ductal adenocarcinoma [129]. Among other mutant cancer driver genes, only TP53 mutants made pancreatic tumor independent of EGFR signaling [129]. An essential role of EGFR signaling in KRAS-driven pancreatic tumorigenesis was also supported by another study, carried out by Ardito and coworkers [130]. These authors showed that oncogenic KRAS upregulates endogenous EGFR activation, a phenomenon dependent on the EGFR sheddase ADAM17 [130]. Importantly, genetic ablation or pharmacological inhibition of KRAS or ADAM17 markedly reduced KRAS-driven tumorigenesis in vivo [130]. Finally, it was shown that in the absence of a sustained EGFR activity, KRAS involves EGFR to induce a robust ERK activation, required for pancreatic tumorigenesis.

In addition to genetic alterations and to the corresponding mechanisms, also epigenetic mechanisms play a relevant role in the genesis of pancreatic cancer. In this context, a relevant role seems to be played by altered activity of histone demethylases. Thus, a recent study provided evidence that the deregulated expression of KDM2B, a member of the histone demethylase family, plays a relevant role in pancreatic cancer pathogenesis. KDM2B was found to be overexpressed in pancreatic cancer, its expression being directly correlated with disease progression [131]. Overexpression of KDM2B cooperated with KRAS oncogenic mutants (KRAS^G12D^) to promote pancreatic cancer formation in mice [131]. Functional experiments provided evidence that KDM2B promoted tumorigenicity through two different transcriptional programs, one consisting of the repression of developmental genes through co-binding with polycomb group proteins at the transcriptional start site and the other consisting of the co-binding with MYC and/or KDM5A, with consequent positive regulation of a set of genes involved in metabolic homeostasis [131].

In conclusion, the studies on the genetic alterations in pancreatic cancer have allowed defining a set of frequently altered genes that can drive the tumorigenic process. In this context, it was evident from these studies that telomere shortening and activating mutations in *KRAS* are among the more frequent and early events in pancreatic carcinogenesis; these gene abnormalities are followed at later stages of tumor development by inactivating mutations of the *p16* (*CDKN2A*) tumor suppressor and in the *TP53* and *SMAD4* tumor suppressor genes. Evaluation of the temporal sequence of these mutations indicated that the large majority of these mutations can be classified as founder mutations, defined as those mutations present at the level of the intraductal precursor lesion in a clonal cell population that later gives rise to the development of the infiltrating carcinoma [17]. In line with this interpretation, a large majority of deleterious mutations were observed at the level of intraductal carcinogenesis [15,17].

On the other hand, some studies have addressed the problem of the molecular mechanisms of the metastatic spreading of pancreatic cancer. In this context, it is important to note that the large majority of pancreatic cancer cells form metastases. The factors responsible for the development of metastases in pancreatic cancer are not completely known. In this context, it was shown that genetic inactivation of *SMAD4* and the subsequent deregulation of canonical TGF-β signaling are highly correlated with the development of metastases [132]. However, 12% of patients had no metastatic disease at autopsy, and an additional 18% had limited metastatic burden [132]. Genetic inactivation of *TP53* or *SMAD4* was significantly associated with the metastatic capacity of pancreatic tumor cells. It is of interest to note that the inactivation of these two genes is often coexistent in the same carcinoma, and this observation prompted analyzing whether there is a peculiar type of *TP53* mutations associating with *SMAD4* loss. This study showed that *TP53* abnormalities due to missense mutations were preferentially associated with *SMAD4* loss, while *TP53* inactivation found in association with wild-type SMAD4 was mainly due to null mutations, including deletions, frameshift or non-sense mutations. Based on these observations, it was suggested that the role of *SMAD4* loss would consist of removing residual apoptotic and cytostatic functions of *TP53* missense mutant proteins.

PDAC is a unique tumor from an immunological point of view due to the presence of several mechanisms that limit the anti-tumor immunological response. Fist, PDACs are characterized by the occurrence of a strong desmoplastic reaction promoting an angiogenetic response and impairing the development of an immune anti-tumor response. Second, PDACs are typically associated with a low burden of tumor infiltrating lymphocytes. PDACs are associated with the massive infiltration of strongly immunosuppressive leukocytes exerting a marked inhibitory effect of an anti-tumor immune response. The mutated RAS oncogene drives an inflammatory reaction that determines a change in the tumor microenvironment promoting a condition of tumor immune privilege. Although mechanisms of immune evasion and immune inhibition are frequent and play an important role in PDAC development, single-agent inhibitors of immune check-points have not demonstrated clear efficacy in PDAC, reviewed in [133]. In line with these observations, PDACs not expressing immunosuppressive markers, such as CD163, FoxP3 and PD-L1, have a prolonged overall survival, compared to the tumors expressing these markers [134].

## 7. Pancreatic Progenitors

The adult pancreas comprises three major lineages: endocrine, acinar and ductal. The endocrine compartment is in the islets of Langerhans and consists of cells that secrete insulin and whose failure to secrete this hormone leads to diabetes. Acinar cells produce digestive enzymes and, together with ductal cells, form the exocrine pancreas. The pancreas develops from part of the foregut endoderm around it. During the primary transition, a pancreas-specified and relatively homogeneous multipotent progenitor cell (MPC) population evaginates from the naive endodermal tube and proliferates to form dorsal and ventral buds. Multipotent pancreatic progenitors proliferate extensively and undergo branching morphogenesis. Later during development, scattered groups of cells undergo apicobasal polarization to initiate the formation of microlumens, which expand and coalesce to build a web-like network (or plexus) of epithelial tubes. The onset of pancreatic specification at about four weeks post-conception in humans is marked by the appearance of PDX1-expressing multipotent progenitor cells. Mounting evidence indicates that Notch-dependent feedback determines the balance between endocrine differentiation and progenitor maintenance of PPCs. Constitutive activation of Notch in Pdx1-expressing epithelial cells blocks differentiation in favor of an undifferentiated progenitor state, whereas Notch inactivation results in “default” acquisition of an endocrine phenotype. Genetic inactivation of the transcription factor and endocrine lineage determinant Neurogenin3 (Ngn3) results in reduced Notch activity and derepression of Ngn3 promoter activity throughout the trunk, suggesting that inhibitory Notch signals are derived from the differentiating endocrine cells [135].

Recent studies indicate an essential role for the transcription factor GATA6 in pancreas organogenesis. In fact, *GATA6* heterozygous inactivating mutations have been identified in patients with pancreatic agenesis, a rare birth defect characterized by complete absence of the pancreas or an extreme reduction of its size; consequently, these patients suffer from severe exocrine pancreas insufficiency and neonatal diabetes. In line with these observations, two recent reports in various experimental systems, including human pluripotent stem cells, support a key role of GATA6 in the induction of human definitive endoderm, development of the pancreas and functionality of exocrine and endocrine pancreas [136,137]. An interaction between GATA6 and GATA4 is required for the generation of pancreatic progenitor cells [136]. GATA6 and GATA4 inactivation in mice determines a dramatic upregulation of Hedgehog components, not compatible with pancreatic determinations [138].

The normal adult pancreatic tissue mainly contains committed progenitors found in pancreatic ducts and in pancreatic glands: these precursors have a limited proliferative and self-renewal activity. Other studies have identified rare cells in the pancreatic tissues displaying the expression of the multipotent transcription factors OCT4 and SOX2: these cells display the property of multipotent stem cells [139]. The phenotype of these progenitors and their contribution to the exocrine and endocrine compartments of the pancreas are highly debated. In spite of this consistent uncertainty, recent genetic lineage tracing experiments have provided evidence that doublecortin-like kinase 1 (DclK1) labels a long-lived rare population of quiescent pancreatic progenitor cells [140]. These Dclk1^+^ cells can proliferate and sustain pancreatic organoid growth, and importantly, these cells play a fundamental role in pancreatic regeneration following tissue injury and chronic inflammatory processes: in fact, their loss had detrimental effects on pancreatic regeneration in these conditions [140].

Recently, glycoprotein 2 (GP2) was identified as a novel cell surface marker for the immature pancreatic progenitor cells derived from pluripotent stem cells [141,142]. In line with these observations, GP2^+^ pancreatic progenitor cells have been identified in fetal human pancreas [143]. GP2^+^ cells generate pancreatic acinar progeny, while GP2^−^ cells generate a ductal and endocrine β-cell progeny [143].

Few data are available about the signals that regulate the epithelial versus endocrine differentiation on multipotent pancreatic progenitors. A recent study identified signaling molecules and transcriptional regulators that drive endocrine cell fate and generation at the level of multipotent progenitor cells [144]. Particularly, evidence was provided that the PDX1-Oc1 interaction is critical at multipotent stages to promote the specification of endocrine progenitors by regulating Neurog3 and other developmentally important genes [145]. A proper expression of Pdx1 and Oc1 in multipotent progenitors is strictly required for differentiation and maturation of β-cells [144].

In addition to these progenitors, a new progenitor cell source for pancreatic cancer stem cells was observed at the level of biliary trees. In fact, at the level of biliary trees, peribiliary glands form stem cell niches for multiple populations of stem cells with indefinite expression potential in vitro and encompassing pancreatic stem cells, islet precursors, hepatocyte and cholangiocyte stem cells, the differentiation potential of these cells being greatly influenced by the tissutal microenvironment [145,146].

## 8. Pancreatic Cancer Stem Cells

Pancreatic cancer stem cells had been initially identified in 2007 by Li and coworkers who investigated the expression of CD44, CD24 and epithelial-specific antigen (ESA) in pancreatic tumors. These authors have isolated CD44^+^CD24^+^ESA^+^ cells from xenografts in immunodeficient mice of pancreatic cancer cells and have shown that these cells are highly tumorigenic and regenerated into host immunodeficient mice the original tumor histology and heterogeneity [147]. A second study by Hermann and coworkers provided evidence that CD133^+^ cells isolated from human pancreatic tumors can initiate a tumor when inoculated into immunodeficient mice [148]. In this study, it was shown also that CD133^+^ cells are resistant to gemcitabine treatment compared to CD133^−^ cells [134]. Furthermore, CD133^+^/CXCR4^+^ cells were shown to be responsible for metastasis development [134]. Other studies have shown that ALDH^+^ cells isolated from pancreatic tumors have the property of cancer stem cells [148]. The presence of ALDH^+^ cells within pancreatic tumors was associated with the clinical and biologic properties of pancreatic cancers. Thus, an increased ALDH expression, as detected by immunohistochemistry in primary localized pancreatic cancers, was associated with reduced overall survival compared to that observed in patients with low ALDH expression [149]. Furthermore, it was observed that ALDH expression was increased in metastatic lesions compared to the primary tumors from the same patient, thus suggesting a role for these cells in tumor metastasis [149]. Finally, it was shown that purified ALDH^+^ cancer stem cells exhibit a mesenchymal phenotype and were highly invasive compared to the bulk tumor [149].

Another study confirmed that ALDH positivity can be used as a marker to identify and to purify pancreatic cancer stem cells. In fact, it was shown that the tumor-initiating cell frequency in the ALDH^+^ cell population was significantly higher (about one in 300 cells) than in the unfractionated cells (about one in 5000 cells) [150].

Kim and coworkers have comparatively studied from the same tumors the tumorigenic potential of ALDH^+^ and CD133^+^ cells. ALDH^+^ cells were consistently shown to be more tumorigenic than CD133^+^ cells [151]. Furthermore, ALDH^high^ cells are markedly more tumorigenic than ALDH^low^ cells [151]. Furthermore, using a triple labeling of the cells, it was shown that ALDH^high^/CD44^+^/CD24^−^ cells are highly tumorigenic, while ALDH^high^/CD44^+^/CD24^+^ cells are scarcely tumorigenic [151]. Finally, it was observed that ALDH expression was highly variable in various pancreatic cancers.

Other studies have focused on the characterization of pancreatic cancer cells expressing the membrane receptor c-met as putative cancer stem cells. C-met is a membrane tyrosine kinase, able to bind as ligand the hepatocyte growth factor (HGF) and, following its activation, stimulates invasion, motility and metastasis. The expression level of c-met in pancreatic cancer tissue is increased compared to normal pancreatic tissue and, in these cells, stimulates cell proliferation. Based on these observations, Li and coworkers have explored the potential role of c-met as a pancreatic cancer stem cell marker [152]. To this end, they have labeled primary pancreatic cancer cells with an anti-c-met antibody and separated the cells into a c-met^high^ and a c-met^low^ population: c-met^high^, but not c-met^low^, cells are able to induce tumor sphere formation in vitro; c-met inhibitors significantly reduced tumor-sphere formation in vitro; c-met^high^ and, particularly, c-met^high^/CD44^+^ cells have an increased tumorigenicity potential when inoculated into immunodeficient mice [152]. Another study explored in detail the effect of c-met inhibitors on pancreatic cancer stem cells. Thus, Hage and coworkers have explored the effect of XL184 (cabozantinib), a c-met inhibitor under clinical evaluation, on pancreatic cancer stem cells. XL184 markedly inhibited tumorsphere formation from c-met^+^ pancreatic cancer cell lines, while it had little effect on normal pancreatic ductal cell [153]. After long-term treatment based on cycles of XL184, pancreatic cancer cells surviving to the treatment had altered apoptotic signaling, but still responded to new cycles of treatment with this drug [154]. Importantly, XL184 in primary cultures of pancreatic cancer stem cells induced the inhibition of the expression of cancer stem cell markers, such as SOX2, c-met and CD133, and induced apoptosis [153]. These observations support the experimental use of c-met inhibitors in the treatment of pancreatic cancer [153]. Thus, cabozantinib was tested in PDAC patients in association with gemcitabine. This drug combination resulted in an excessive clinical toxicity; three out of 10 evaluable patients displayed a partial response [155]. Therefore, these data indicate that this drug combination is impractical for further development, due to excessive toxicity [155]. It was recently proposed that the combined inhibition of Hedgehog and c-Met pathways could represent a useful therapeutic strategy, for their synergistic anti-tumor activity and for their capacity to bypass drug resistance occurring after single-drug treatment [156].

The side population (SP) technique was used to evaluate its potentiality to identify pancreatic cancer stem cells. As is known, the SP technique identifies cells that can expel Hoechst-dye, due to the presence of multi-drug resistance transporters. In many cancer cell types, the SP is enriched in cells exhibiting properties of cancer stem cells. Van den Broeck and coworkers have explored the SP in primary pancreatic cancer samples showing that: SP cells are detectable in all primary tumors, at variable proportions; isolated SP cells are more resistant to gemcitabine than the bulk tumor; whole-genome expression profiling of the SP demonstrated the expression of genes involved in cancer pathways, particularly in chemoresistance and EMT; the multidrug transported ABCG2 is highly expressed in SP cells; SP cells are tumorigenic both in vitro and in vivo [157].

The methodology for standard growth in vitro of cancer stem cells implies that tumor cells are grown under serum-free conditions, using growth factor combinations that favor the growth of stem-like cells. Under these cell culture conditions, three types of tumor spheres are formed: holoclones, meroclones and paraclones, with holoclones being smaller, round and composed of small and tightly-packed cells with regular and smooth colony borderlines; paraclones were composed of dispersed and larger cells with fragmented borderlines; meroclones exhibited an intermediate morphology [143]. The different types of colonies possessed differential capacities for self-renewal and long-term proliferation, and particularly, only holoclones initiate tumor formation and supported tumor serial transplantation in NOD/SCID mice [157]. The expression of various stem cell-associated markers was higher in holoclones than in other types of colonies [143]. Finally, holoclones were much more chemoresistant than paraclones and meroclones [157].

Several recent studies have attempted to define some transcription factors that play a key role in the regulation of pancreatic CSCs. Among the various factors, the polycomb factor Bmi1 seems to play an important role. Bmi1 was found to be overexpressed in PanIN lesions, pancreatic adenocarcinomas and pancreatic cancer cell lines. This observation, as well as the role of this factor in the proliferation of pancreatic progenitors, as well as its role in some animal models of pancreatic tumorigenesis stimulated the investigation of the mechanisms through which this factor could promote pancreatic cancer development. In this context, Bmi1 was found to be overexpressed in the cancer stem cell compartment of primary human pancreatic cancer xenografts; in line with this observation, pancreatic tumorspheres were shown to possess high Bmi1 levels [158]. Silencing of Bmi1 greatly decreased tumor growth of primary tumor xenografts, considerably inhibited the tumorigenic activity of secondary and tertiary tumorspheres and significantly reduced the number of cancer stem cells at the level of xenograft tumor tissue [158]. These observations suggest an important role for Bmi1 in the control of pancreatic CSCs [158]. Another polycomb family protein, E2HZ, was shown to play a relevant role in the maintenance of pancreatic cancer stem cells. E2HZ was found to be highly expressed in enriched CSC populations isolated from pancreatic cancer cell lines; on the other hand, E2HZ knockdown by RNA interference significantly reduced the number of pancreatic CSCs [159].

A recent study explored two stem cell markers of many tissutal compartments, LGR5 and Nanog, as potential markers for pancreas CSCs. Thus, it was shown that LGR5, a marker of intestinal stem cells, is expressed in normal pancreas at the level of the islets of Langerhans, where it is co-localized with Nanog and insulin in clusters of β-cells [160]. In pancreatic cancer tissue, LGR5 and Nanog staining is observed at the level of the remaining islets and in ductal cancer cells [160]. According to these findings, it was suggested that the islet’s β-cells expressing LGR5 and Nanog are the initiating cells of pancreatic cancer, which migrated from the islets to form the ductal tissue, after complex mutations and de-differentiation [160]. The important role of Nanog in the stemness of pancreatic cancer cells was supported also by other recent studies. Thus, Lu and coworkers have provided evidence that high Nanog and Oct4 expression in primary pancreatic cancer had a negative prognostic impact, and Nanog and Oct4 knockdown in CSCs isolated from prostate cancer cell lines resulted in a reduction of proliferation, invasion, chemoresistance and tumorigenesis [161].

A recent study explored the role of urokinase plasminogen activator (uPA) in the development and maintenance of pancreatic cancer stem cells. Particularly, it was shown that the suppression of the expression of uPA in pancreatic CSCs markedly reduced the tumorigenicity and chemoresistance of these cells. Other experiments have shown that uPA promotes the stemness of pancreatic cancer cells through a mechanism involving the direct interaction of uPA with the transcription factors HOXA5 and Hey: particularly, uPA regulates Lhx-2 expression by suppressing expression of miR-124 and p53 expression by repressing its promoter by inactivating HOXA5 [162]. These observations indicate that regulation of gene transcription by uPA contributes to pancreatic cancer stemness [162].

Bao and coworkers have isolated a population of triple-marker-positive cells (CD44^+^/CD133^+^/EpCAM^+^) enriched in cancer stem cell-like cells from two pancreas cell lines and have shown that these cells preferentially express, compared to triple-marker-negative cells, some genes including BMi1, BMP4, BST2, BTG1, FolR1, FoxQ1, PRKAR1A, Sox4, TACTD2 and Wnt3a [163]. Among these genes, FoxQ1 was found to be relevant and required for the aggressive biological properties of these cells: in line with these findings, FoxQ1 knockdown decreased EpCAM and Snail expression in pancreas CSC-like cells [163]. Using the same experimental approach, these authors have also shown that triple-positive cells isolated from pancreatic cancer lines display a peculiar pattern of expression of several miRNAs: among them, miR-125b was found to be overexpressed in triple-positive cells, and its knockdown elicited a marked inhibition of tumor aggressiveness of these cells, consistent with the downregulation of CD44, EpCAM and EZH2 [164].

CD44 seems to be one of the most reliable markers of pancreatic cancer stem cells. Recent studies have explored the mechanisms responsible for CD44 expression in PDAC [165,166]. Particularly, two studies provided evidence that the FOXO3 transcription factor is essential for CD44 expression in pancreatic cancer cells [165,166]; particularly, a complex signaling/metabolic pathway involving FOXO3/c-AMP/peroxisome proliferator-activated receptor-γ co-activator-1β (PCG-1β)/pyruvate dehydrogenase-A1 is essential for CD44 expression and cancer stem cell properties in pancreatic cancer cells [166]. The 67 laminin receptor (67LR) is overexpressed in various cancers, including PDACs. In PDACs overexpressing 67LR, a phosphodiesterase inhibitor (PDE3) and epigallocatechin 3-*O*-gallate (EGCG) in combination significantly suppressed the FOXO3-CD44 axis in pancreatic cancer stem cells [167].

Recent studies characterized Dclk1^+^ cells as candidate pancreatic CSCs. As mentioned above, a recent study provided evidence that DCLK1^+^ cells may act as multipotent pancreatic progenitors. Increased DCLK1 expression was reported in both PanINs and PDACs [168]. Initial studies have provided preliminary evidence that both pre-invasive and invasive pancreatic cancers depend for their growth on DCLK1-positive cells with CSC properties and that these cells highly express ABL1 and IGF1R, both required for their oncogenic properties [124,169,170]. Using a model of liver invasive pancreatic cancer, Ito and coworkers showed that DCLK1^+^ cells were predominantly expressed at the level of cells with CSC properties: these cells were highly metastatic and preferentially localized at the level of invading tumor margins [171]. Overexpression of DCLK1 increased the tumorigenic properties, while DCLK1 knockdown decreased the metastatic activity of CSCs [157]. Westphalen and coworkers have explored the tumorigenic potential of DCLK1^+^ cells in experimental mouse models providing evidence that: (a) the simple introduction of mutant KRas into DCLK1^+^ cells does not modify the proliferation, survival or longevity of these cells; (b) in contrast, the introduction of a mutant KRAS, together with an inflammatory stimulus (pancreatitis), converts DCLK1^+^ cells into potent cancer-initiating cells [140]. According to these findings, it was concluded that DCLK1, as a potential KRAS effector, functionally contributes to the pathogenesis of pancreatic cancer [140]. Forty eight percent of primary PDACs were positive for DCLK1, and DCLK1^+^ tumors had significantly shorter survival times than DCLK1^−^ tumors (19 tumors vs. 49 months) [171]. DCLK1 expression correlated in PDACs with EpCAM expression [172].

In parallel with the study of cancer stem cells, other investigations were focused on trying to develop an efficient xenotransplantation assay of primary PC tumors. The generation of patient-derived tumor xenografts has proven useful for the screening of new drugs and the discovery of new biomarkers, thus contributing to the discovery of fundamental information on tumor biology and on the potential efficacy of new drugs and to the personalization of anti-cancer treatments. The xenotransplantation assays have been performed using two different routes of inoculation of cancer tissues: subcutaneous xenotransplantation; orthoptic xenotransplantation.

Rublo-Viqueira and coworkers first reported the xenotransplantation model of pancreatic cancer. In their experimental approach, they implanted 3 × 3 × 3 mm^3^ of pancreatic cancer tumor tissue imbedded into Matrigel subcutaneously into nude mice [173]. However, these authors showed that the pattern of gene expression of xenotransplants does not reflect with fidelity the gene expression profile of primary tumors [173]. Furthermore, the subcutaneous transplantation model failed to metastasize at the level of the peritoneum and of the liver [173]. Garcia and coworkers have reported an efficient methodology to obtain the frequent engraftment (85% of cases) of primary pancreatic cancer tissue fragments into SCID mice (tumor tissue was implanted at the base of the tail) [174]. The tumor xenotransplants can be propagated into secondary and tertiary mice and exhibit pathological and molecular features like the tumors from which they were derived [174]. The 15% of cases unable to engraft immunodeficient mice have either a low percentage of tumor cells or have a low tumoral mass [174]. Wennerstrom and coworkers have reported the successful growth of primary pancreatic cancer specimens in NOD/SCID mice IL-2Rγ^null^ (NSG mice) through subcutaneous injection of tumor cells [175]. The xenotransplanted tumors have been used for the development of new human pancreatic cancer cell lines [175]. Other investigators have developed an orthoptic model of pancreatic cancer xenotransplantation, where primary tumoral tissue is implanted at the level of the pancreas. Using such an approach, Walters and coworkers reported a different rate of successful implantation following using primary pancreatic tumors (38%) or metastatic tumors (88%) for implantation [176]. A peculiarity of this model is that the xenotransplanted tumors form peritoneal and hepatic metastases [176]. Delitto and coworkers reported the successful generation of 15 PDXs from 25 primary tumor specimens and showed that the xenografts conserved the original histology of the tumors from which they were generated [177]. Importantly, mouse stromal cells infiltrated the human cancer cells, suggestive of active tumor-stromal cells in these tumor models [162]. The mutational profile of the xenografts seems to be maintained during serial passages [178]. More recently, Jung and coworkers reported the successful growth in 72% of cases of primary human pancreatic cancer specimens injected subcutaneously into NOD/SCID mice [179]; importantly, the xenografts conserved a pattern of TP53 and SMAD4 expression like the original tumor [179]. Finally, the correspondence of the mutational profile of the pair’s primary tumors and corresponding xenografts was good [179].

One of the main reasons for the development of xenotransplantation models of human pancreatic cancers consists of the use of these models as biological platforms to evaluate new drugs. In this context, various studies have been reported in the literature. It has been pointed out that xenotransplantation tumor models of pancreatic cancer have a great limitation concerning their use for drug evaluation. In fact, cell line-based, but also primary tumor-based xenografts grow as masses of cancer cells with minimal (for cancer cell lines) or low (for primary tumors) stromal infiltration: this reduced desmoplastic reaction observed in xenotransplant models leads to overestimation of the effect of various anti-tumor drugs [180]. The importance of the stroma in pancreatic cancer is strongly supported by the observation that the engraftment of primary pancreatic adenocarcinomas is associated with the expression of stromal gene pathways and decreased patient survival [181]. Furthermore, patients with engrafting tumors have more frequent SMAD4 loss [181].

Using the orthoptic pancreatic cancer xenograft model mentioned above, two new drug combinations were tested. In a first study, the combination therapy with trametinib, an MEK1/2 inhibitor, and lapatinib, an EGFR and HER2 activity inhibitor, was tested; in four of five patient-derived xenotransplants, the drug combination elicited a marked growth inhibition [182]. In a more recent study, the same authors have evaluated the drug combination of trametinib with panitumumab (MoAb inhibiting EGFR) and trastuzumab (Mab inhibiting HER2): these three-drug combinations were more active than the two-drug combinations reported above at inhibiting pancreatic cancer xenotransplants [183]. Interestingly, Hermann and coworkers have used a combined experimental approach using both tumor-spheres and tumor xenografts to evaluate the anti-tumor activity of a drug combination involving gemcitabine, a Hedgehog and an mTOR inhibitor [184]. This drug combination could eliminate CSCs and led to long-term survival in primary human pancreatic cancer tissue xenografts [184]. Using PDXs isolated from 12 PDACs, it was shown that phenformin treatment elicited higher anti-tumor efficacy than other metabolic inhibitors and metformin [185]. These observations warrant further evaluation of phenformin as a new therapeutic agent in PDAC [185].

Another recent study suggests that CD47 could represent a potentially interesting target for targeting of CSCs also in PDACs. CD47 was highly expressed in CSCs, but not in other nonmalignant cells in the pancreas. Targeting CD47 efficiently enhanced phagocytosis of a representative set of primary human pancreatic cancer (stem) cells and, even more intriguingly, also directly induced their apoptosis in the absence of macrophages during long-term inhibition of CD47. In patient-derived xenograft models, CD47 targeting alone did not result in relevant slowing of tumor growth, but the addition of gemcitabine or Abraxane resulted in sustained tumor regression and prevention of disease relapse long after discontinuation of treatment. These data are consistent with efficient in vivo targeting of CSCs and strongly suggest that CD47 inhibition could be a novel adjuvant treatment strategy for PDAC independent of underlying and highly variable driver mutations [186]. Interestingly, CD47 was involved also in the mediation of the anti-tumor activity of human exosomes engineered with siRNA or short hairpin RNA targeting KRAS^G12D^: following injection into immunocompetent mice, these exosomes exhibited superior evasion of phagocytic clearance, compared to lysosomes, due to the expression on their surface of CD47, whose presence is required to mediate exosome escape from phagocytosis [187]. In fact, CD47-knockout exosomes exhibited a clearly reduced half-life in serum [187]. Interestingly, in orthoptic KRAS^G12D^ tumors, engineered exosomes were found to accumulate preferentially in tumoral areas, compared to normal adjacent pancreas.

Studies based on the inhibition of regulatory pathways that are essential for the self-renewal capacity of pancreatic CSCs are promising; however, the overly heterogeneous genetic background of PDAC may render larger populations of cells resistant to the targeting of single pathways. Studies carried out by Lonardo and coworkers have shown that Nodal and Activin belonging to the TGF-β superfamily regulate self-renewal of pancreatic cancer stem cells [188]. Nodal and Activin were hardly detectable in more differentiated pancreatic cancer cells, while cancer stem cells and stroma-derived pancreatic stellate cells markedly overexpressed Nodal and Activin. Knockdown or pharmacological inhibition of the Nodal/Activin receptor Alk4/7 in cancer stem cells virtually abrogated their self-renewal capacity and in vivo tumorigenicity and reversed the resistance of orthotopically engrafted cancer stem cells to gemcitabine. However, engrafted primary human pancreatic cancer tissue with a substantial stroma showed no response due to limited drug delivery. The addition of a stroma-targeting Hedgehog pathway inhibitor enhanced delivery of the Nodal/Activin inhibitor and translated into long-term, progression-free survival. Therefore, inhibition of the Alk4/7 pathway, if combined with Hedgehog pathway inhibition and gemcitabine, provides a potentially valuable therapeutic strategy for targeting cancer stem cells [188]. Human PDACs contain mutations of activing receptor type 1B; these mutations seem to have an oncogenic role since loss of activing receptor type 1B accelerates development of IPMNs in mice with activated KRAS [189].

Recent studies have addressed a considerable interest in the analysis of circulating tumor cells, present in the blood of patients with malignant tumors, as a diagnostic and prognostic tool. These studies have shown that circulating tumor cells (CTCs) are present in the blood of patients with various types of solid tumors, including PDACs [190]. Interestingly, a recent report provided evidence that CTCs isolated from the blood of PDAC patients display cancer stem cell markers [191]. Particularly, 78% of PDAC patients displayed CTCs expressing, in addition to an epithelial marker (cytokeratin), also a cancer stem cell marker (ALDH, CD133 or CD44) [191]. The presence of cytokeratin^+^/ALDH^+^ or cytokeratin^+^/ALDH^+^/CD133^+^ cells was associated with a worse prognosis and was a predictor of tumor recurrence [191].

In addition to offering the opportunity to detect circulating tumor cells, the peripheral blood of PDAC patients represents also a potential source of circulating tumor DNA (ctDNA). In cancers like PDAC, the presence of ctDNA is of paramount importance, given the considerable difficulty in obtaining tumoral tissues from patients using endoscopic techniques and because only about 20% of patients present a resectable tumor. Initial studies have shown that ctDNA can be isolated from most PDAC patients and together with detection of the serum protein CA 19-9 allows performing a diagnosis of PDAC with a sensitivity of 91% [192]. Recent studies have shown the prognostic value of ctDNA: the presence of ctDNA was associated with a poor prognosis [192]; furthermore, the presence of ctDNA among PDAC patients who had curative intent resection is associated with a shorter progression free survival (PFS) and OS [193]. The combination of ctDNA detection, together with protein biomarkers (CA 19-9, CEA, HGF, OPN), was shown to be useful for the early detection of PDACs [194]. Finally, in PDAC patients, changes in ctDNA levels of mutant KRAS can be used as a sensitive marker of response to chemotherapy regimens [195].

CA 19-9, which is currently in clinical use as a PDAC biomarker, has limited performance in detecting early-stage disease. Protein biomarker candidates that have the potential to complement CA 19-9 have been identified [196,197]. Some sets of these new serum biomarkers, together with CA19-9, improved the detection of early-stage pancreatic cancer [196,197].

## 9. Mechanisms of Matricellular Fibrosis in Pancreatic Cancer

PDAc is characterized by a dense fibrous stroma (desmoplastic stoma) composed of numerous cell types including pancreatic stellate cells (PSG) or carcinoma-associated fibroblasts (CAFs) and macrophages and extracellular matrix components, including fibrous proteins, such as collagen, glycoproteins, such as fibronectin, and polysaccharides, such as hyaluronic acid. This stromal reaction in PDACs is massive, and transformed epithelial cells represent only a minority of the tumoral mass. This tissutal desmoplastic reaction to the growth of neoplastic cells is relevant to pancreatic tumorigenesis, but its exact role is controversial since some studies suggest a pro-tumorigenic role, while other studies indicate an inhibitory role on pancreatic tumorigenesis.

In mouse models of PDAc tumorigenesis, the inhibition of the capacity of pancreatic tumor cells to respond to Hedgehog peptides induces a reduction of the stromal component of PDACs, associated with an increase of tumor aggressiveness; in contrast, Hedgehog activation in PDAC tumors using small-molecule agonists increased tumor fibrosis and decreased tumor growth [198]. On the other hand, depletion of fibroblasts at the level of experimental PDAC tumors increased tumor growth and progression [199]. In spite of the indications derived from these studies, anti-stromal therapies in clinical trials have failed to show the expected benefits.

Recent studies have more directly investigated in human PDACs the possible contribution of the stromal component to tumor growth and a possible link with genotypic features of these tumors. Thus, Laklai and coworkers observed that human PDACs with impaired TGF-β signaling (characterized at the molecular level by SMAD4 inactivation) have high STAT3 activity and develop stiff, matricellular-enriched fibrosis (increased collage-fiber diameters), associated with high epithelial tension and shorter patient survival [39]. These observations were supported by the study of animal models based on KRAS-driven tumorigenesis, associated with loss of TGF-β signaling, reproducing the phenotype observed in primary human tumor samples and supporting also the role for STAT3 as an inducer of the tissutal fibrotic response [39]. According to the results of this study, it was concluded that PDAC-associated fibrosis cannot be considered only a physical barrier that reduces drug delivery to neoplastic tissue, that decreases the accessibility of immune cells to the tumor and promotes drug resistance, but also a force driving an elevated tissue mechanics, involved in the activation of important pathways, such as integrin-dependent signaling and YAP activation [39]. Therefore, since biomechanical forces can drive tumor aggression by inducing a mesenchymal-like switch, strategies that could reduce tumor mechanics may represent effective approaches to prevent the emergence of treatment-resistant pancreatic cancers [200].

Other studies have highlighted the important role of the interaction between tumor cells and stromal cells as a mechanism inducing activation of some stromal cells, thus acquiring properties necessary to sustain tumor growth. Thus, the interaction of PDAC cells with CAFs induces in these cells the methylation of some genes and, notably, of SOCS1 [200]; the inhibition of SOCS1 in fibroblast cells activates STAT3 and induces the release of insulin-like growth factor-1, required to sustain the growth of pancreatic cancer cells [201].

Other studies have shown the key role of focal adhesion kinase 1 (FAK1) as an important mediator of PDAC fibrosis. FAK1 is hyper activated in the large majority of PDACs, and its level of expression is correlated with an immunosuppressive tumor microenvironment (low number of CD8^+^ T lymphocytes and high number of granulocytes [201]); importantly, high FAK1 expression was associated with high levels of fibrosis [202]. In line with these observations, FAK inhibitors decreased tumor growth and progression in mice PDAc models; furthermore, FAK inhibitors increased the sensitivity of PDAC cells to immunotherapy and render unresponsive tumors responsive to immunotherapy [202].

In addition to pancreatic stellate cells and fibroblasts, also tissutal macrophages play a key role as mediators of fibrosis. Studies on experimental murine models of PDAC indicate that tumor macrophages are of a heterogeneous origin, with a predominant population represented by tissutal macrophages of embryonic origin, displaying a pronounced fibrogenic activity and exhibiting a pro-fibrotic transcriptional profile [203].

## 10. Neuroendocrine Pancreatic Tumors

As stated in the Introduction, in addition to PDAC, largely the most frequent pancreatic tumor, there are less frequent types of pancreatic tumors. Among them, the most frequent are pancreatic neuroendocrine tumors (PanNET). There is a sporadic (more frequent, 90% of cases) and a familial form of PanNET (more rare, 10% of cases). PanNETs have a different degree of malignancy and when displaying a Ki67 index more than 20% are classified as pancreatic neuroendocrine cancers (PanNEC). The World Health Organization classification, based on the assessment of the proliferative fraction of PanNET tumors, divides these tumors into three groups: low-grade, intermediate-grade and high-grade. G3 tumors represent about 10% of the total and are tumors that are invariably lethal; G1/G2 tumors display a highly variable clinical course, ranging from indolent to highly malignant. Whole exome sequencing studies have shown that sporadic PanNETs display an average of 16 somatic mutations, with somatic MEN1 mutations being present in 41% of these tumors [203]. In addition to MEN1 mutations, sporadic PanNET displayed in 45% of cases inactivating mutations of *ATRX* or *DAXX* and 15% in mTOR pathway genes [204]. Interestingly, *ATRX* or *DAXX* loss was associated with telomere alterations (i.e., the alternative lengthening of telomeres, a mechanism of telomere maintenance) [184], chromosome instability and reduced survival [205]. Gene gains and losses are also frequent in sporadic PanNET: VHL is deleted in 18% of patients; allelic loss of *PHLDA3*, a regulator of mTOR pathway, was observed in 70% of patients [206].

**Figure 5:**
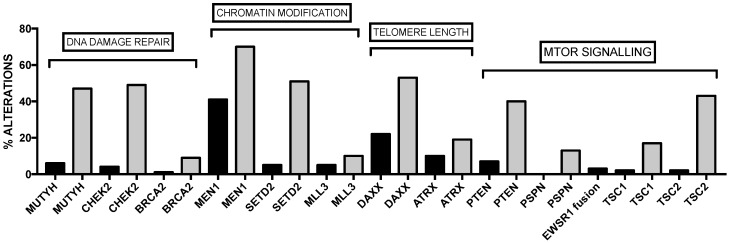
Frequency of the main genetic abnormalities observed in pancreatic neuroendocrine tumors (PanNET), subdivided into biochemical pathways. For each gene are reported the mutations (black bars) and copy number alterations (grey bars).

A recent study reported an extensive characterization of genetic alterations occurring in sporadic PanNETs, based on whole-genome sequencing of 102 primary sporadic PanNETs [207]. PanNETs displayed a mutational burden lower than PDACs (0.82 somatic mutations per megabase, compared to 2.64 per megabase in PDACs) [206]. An important feature of PanNETs is that these tumors display a larger-than expected proportion of germline mutations, including mutations in the DNA repair genes *MUTYH*, *CHEK2* and *BRCA2*, as well as in MEN1 and VHL, these mutations occurring in 17% of patients [206]. The most recurrent driver mutations included those occurring at the level of the genes *MEN1* (37%), *DAXX* (21%), *ATRX* (11%), *PTEN* (7%), *DEPDC5* (2%), *TSC1* (2%), *TSC2* (2%), *TP53* (3%) and *SETD2* (5%) [207] (Figure 4). Copy number analysis showed that PanNET patients can be subdivided into four groups: recurrent pattern of whole chromosome loss (RPCL); limited copy number events, mostly losses affecting chromosome 11; polyploidy; aneuploidy. The RPCL subtype exhibited loss of specific chromosomes and was enriched in G2 PanNETs [207]. The polyploid group was characterized by the highest somatic mutation rate (1.98 somatic mutations per megabase) [207]. Structural chromosomal rearrangements are less common in PanNETs (29 events per tumor) than in PDACs (119 events per tumor). Some of these rearrangements lead to inactivation of tumor suppressors, such as *MTAP*, *ARID2*, *SMARCA4*, *MLL3*, *CDKN2A* and *SETD2*, or to oncogenic gene fusions (Figure 5). Among the 66 somatic in frame gene fusions observed, notable are three fusion events leading to *EWSR1-BEND2* or *EWSR1-FLI1* [207].

Telomere alterations were observed in *ATRX* or *DAXX* mutant tumors: biallelic inactivation of *ATRX* or *DAXX* through loss of heterozygosity was strongly associated with an increase in telomere length; *MEN1* somatic mutations were associated with increased telomere length [206]. RNA sequencing studies supported the subdivision into three expression subtypes: insulinoma, intermediate and metastasis-like, in line with other studies [208].

The integrated analysis of the main cancer pathways showed that four pathways are commonly altered by mutations in PanNETs: (i) DNA damage repair, involving germ line-damaging variants of *MUTYH*, *CHECK2* and *BRCA2*, globally observed in 11% of patients; (ii) chromatin remodeling, involving recurrent mutations of *MEN1*, *SETD2*, *ARID1A* and *MLL3* genes, that determines a wide deregulation of gene transcription; (iii) telomere maintenance, deregulated by the mutations occurring at the level of ATRX, DAXX and MEN1 genes; (iv) mTOR signaling activation, involved in the cases displaying inactivating mutations in negative regulators of mTOR signaling (*PTEN*, *TSC2*, *TSC1* and *DEPDC5* genes, globally observed in 12% of patients) [207]. In the G2 subgroup, tumors displaying *ATRX* or *DAXX* or inactivating mutations of negative regulators of mTOR are associated with poor prognosis [207].

Other recent studies have confirmed in a large series of PanNET patients that alternative lengthening of telomeres (ALT) and loss of DAXX/ATRX predict metastatic disease and poor survival in patients with PanNET [209,210]. The disease-free survival and overall survival of patients with ALT or DAXX/ATRX is clearly lower, compared to wild-type DAXX/ATRX PanNETs [209,210]. According to these observations, it was concluded that ALT and DAXX/ATRX loss in PanNET was associated with reduced survival and seems to play an important role in driving metastatic disease [209,210].

The genetic alterations occurring in well-differentiated PanNETs have been compared to those observed in PanNECs, providing evidence that these two tumors are genetically comparable, but clearly distinct: in fact, in PanNECs, *KRAS* (about 30%) and inactivating *TP53* (about 60%) and *RB1* (about 70%) mutations are frequent, while they are absent in PanNETs; furthermore, at variance with PanNETs, all PanNECs retained DAXX and ATRX [211]. In the familial PanNETs, a sequence of hyperplasia-neoplasia is observed. MEN1 syndrome associated with PanNET displaying loss of the WT *MEN1* allele is observed in 100% of cases: loss of the WT *MEN1* allele is observed in micro-adenomas, thus implying that *MEN1* loss is an early event. The same conclusion was recently reached also for sporadic PanNET: aberrant MEN1 expression was observed in 74% of micro-adenomas; in contrast, none of these micro-adenomas display the alternative lengthening of telomeres phenotype and do not display DAXX and ATRX loss [212]. All these observations strongly suggest that loss of *MEN1* is an early event in pancreatic neuroendocrine tumorigenesis [212]. Recent studies indicate that loss of DAXX/ATRX expression and alternative lengthening of telomeres predict metastatic disease and poor survival in PanNET patients [209,213]. As mentioned above, PanNETs are characterized by the alternative lengthening of telomeres. In tumor cells, other mechanisms are used for telomerase re-activation/expression, such as telomerase promoter mutations. TERT promoter mutations have been detected in PanNETs, but are associated only with hereditary syndromes and not with sporadic cases [214].

Tumor-initiating cells and the biological processes that promote pathogenesis remain largely uncharacterized in PanNETs. A recent study showed that MET proto-oncogene activation is important for tumor growth in PanNET xenograft models: this approach allowed the identification of a highly tumorigenic cell population within several independent, surgically acquired PanNETs, characterized by increased cell-surface protein CD90 expression and aldehyde dehydrogenase A1 (ALDHA1) activity, displaying in vitro and in vivo evidence for stem-like properties. Proteomic profiling of 332 antigens in two cell lines and four primary tumors was performed, showing that CD47, a cell-surface protein that acts as a “don’t eat me” signal co-opted by cancers to evade innate immune surveillance, is ubiquitously expressed. Moreover, CD47 coexpresses with MET and is enriched in CD90(hi) cells. Furthermore, blocking CD47 signaling promotes engulfment of tumor cells by macrophages in vitro and inhibits xenograft tumor growth, prevents metastases and prolongs survival in vivo [215]. Interestingly, a recent study provided evidence that PanNETs diffusely and robustly express the cancer stem cell marker DCLK1; DCLK1-overexpressing PNET cells exhibited epithelial to mesenchymal characteristics, highlighted by high expression of the SLUG factor; SLUG expression in the cells is controlled by p-FAK [216].

## 11. The Difficulty of Defining Efficacious Medical Therapies for Pancreatic Cancer and Conclusions

PDAc is a great health problem, with an estimated 367,000 new cases diagnosed worldwide in 2015 and an associated 359,000 deaths in the same year. Given these data, it is not surprising that PDAC is currently the fourth highest cause of cancer death in developed countries. If the treatment outcomes of PDAC are not improved, it is estimated that PDAC could become the second cause of cancer-related mortality within the next decade [4]. Many factors impede a rapid progress in PDAC treatment due to the absence of specific biomarkers for an early diagnosis, the aggressive nature of PDAC with rapid local invasion and early metastases, the limited number (15–20%) of PDAC patients amenable to curative surgical resection and the consistent resistance to most treatments, including standard chemotherapy, radiotherapy, molecular targeted therapy and immunotherapy. Furthermore, nonmalignant cells in the tumor microenvironment contribute to anticancer drug resistance. Finally, recent studies indicate that intratumor bacteria, frequently (76% of cases) observed in PDAC patients, contribute to tumor resistance to the chemotherapeutic drug gemcitabine, currently used in the treatment of PDAC [217].

In spite of this impasse in the improvement of PDAC therapy, considerable progress has been made in the last few years in our understanding of PDACs. *KRAS* mutations are sufficient to initiate premalignant lesions, PanINs; PanINs can progress to locally invasive or metastatic cancer by way of either genomic rearrangements or stepwise acquisition of mutations in suppressor genes (*CDKN2A*, *TP53* and *SMAD4*). Genome-wide studies have identified additional mutations and pathways, chromosome alterations, ubiquitin proteases and transcription factor alterations that cooperate with KRAS to drive PDAC progression. Furthermore, analysis of gene expression allowed proposing a classification of PDACs into four different groups, defined as squamous, pancreatic progenitor, immunogenic and aberrantly differentiated endocrine exocrine (ADEX); each of these subtypes is characterized by a different landscape of genetic alterations, tumor histological characteristics and different prognosis. Unfortunately, many of the molecular abnormalities identified in PDAC are not amenable to an efficacious pharmacologic targeting. When targeting therapies are available for PDAC, these therapies have not given satisfactory results; this is due to rapid upregulation of compensatory alternative pathways removing the anti-tumor effect of targeting agents and to the tumoral fibrotic desmoplastic reaction.

The studies of next generation sequencing techniques with whole-genome or exome sequencing offer the opportunity to identify, in some PDAC patients, genetic alterations suitable for therapeutic targeting. However, the practical utility of these studiers has been limited in the ensemble of PDAC patients and, particularly, in those with advanced PDAC, because these methods remain costly and are still relatively rare in a clinically-certified setting. Furthermore, the depth of coverage for these methods is insufficient to allow the detection of relevant genetic alterations in a neoplasm like PDAc, characterized by a paucity of tumoral elements embedded in a predominant stroma.

The current and emerging treatments of metastatic cancer are briefly summarized in Table 2. This table reports only the main treatments under investigation in PDAC patients.

Only very few studies have evaluated the impact of the mutational load and of the type of mutations on PDAC prognosis. In this context, particularly interesting are the results of a study recently published showing that patients with only one mutation among the four more frequently mutated genes (KRAS, TP53, CDKN2A and SMAD4) have a clearly better prognosis than those with two or more mutations; KRAS-wild type PDAC patients have a clearly better prognosis than *KRAS*-mutated patients; finally, *CDKN2A* intact PDACs have a significantly better prognosis than *CDKN2A*-mutated PDACs [217]. Interestingly, in this, it was shown also that PDACs histologically classified as combined with a cribriform component survived longer than patients with other histological features [218].

A recent report evaluated the potential and actual therapeutic implications of comprehensive genetic analysis of patients with PDAC [218]. This analysis was based on a hybridization capture-based, next-generation assay design for deep sequencing of all exons and selected introns of 401 cancer-associated genes [218]. The median time from protocol consent to reporting of the genomic results was 45 days with a median time from tissue delivery of 20 days [219]. This study identified potentially actionable findings in 26% of cases. Five-point-five percent 5.5% of these patients displayed somatic alterations classified as 2b, defined as an approved biomarker in another cancer indication and included *ERBB2* amplifications, *CDK4* amplifications, *BRCA1/2* mutations, BRAFV600E mutation and fusion events involving *ROS1* and *ALK1* [219]. Four-point-six percent of patients displayed 3b genetic alterations, defined as alterations for which clinical evidence links the biomarker to drug response in patients, but use of the biomarker is not standard-of-care, which includes *AKT1* mutations, *ERBB2* mutations, *PI3KCA* mutations and *FGFR1* amplifications [219]. Although this study suggests a possible practical application of next-generation sequencing as a strategy to guide individual patient treatment, its routine use is currently limited in PDAC patients. Future perspective molecular profiling should seek to incorporate routine germline genetic analysis and the identification in tumor biopsies of DNA profiles predicting for clinical benefit from an agent that targets DNA damage repair and/or immunotherapy.

A part of PDAc occurs in families with two or more affected first-degree relatives (familial pancreatic cancers). In a significant proportion of these patients, inheritance was attributed to a set of gene mutations affecting DNA repair genes such as *BRCA1*, *BRCA2*, *ATM* and *PALPB2*. These defects defined the so-called “unstable” PDAC subtype described by Waddell et al. and characterized by a higher mutational load than other PDACs, by numerous defects in genes involved in DAN maintenance and BRCA mutational signature [219]. It was estimated that about 24% of PDACs display a DNA damage response deficiency (DDR) due in 7% of cases to germline mutations of either *BRCA1* or *BRCA2* or *PALB2*, in 7% to somatic mutations of these genes and in the remaining patients to rare mutations of genes such as *ATM*, *RPA1*, *REV3L*, *XRCC4*, *XRCC6* [220]. The PDAC patients with a DDR deficiency are candidates for clinical trials with drugs targeting DDR deficiency [220]. Defective homologous recombination (HR)-mediated DNA repair as a result of mutations in *BRCA1/BRCA2* genes is related to genomic instability and generates a unique sensitivity in cancer cells to DNA-targeting agents, which induce irreversible DNA damages. Preclinical studies have provided evidence that PARP inhibitors abrogate DNA repair in HR-defected cancer cells and inhibit the proliferation of these cells, including PDACs [221]. Clinical studies have provided preliminary evidence that PARP inhibitors and platinum-based compounds may have a significant anti-tumor effects in BRAC-mutant PDACs [220]. However, there is limited evidence that other genes involved in DDR deficiency could benefit from these treatments. In this context, a recent study showed that ATM-mutant PDAC cells are sensitive to PARP inhibitors and to ATR inhibitors, both of these drugs reducing the proliferation and viability of these cells [222]. Importantly, a deficiency of the serine/threonine ATM increases genomic instability and metastatic potential of a mouse model of pancreatic cancer [223]. It is of interest that in parallel to progress in the definition of response to drugs of DRR-deficient PDAc cells, the mechanisms through which mutations of the DNA damage repair induce pancreatic tumorigenesis have been in part elucidated. Thus, it was shown that BRCA2 mutation induces pancreatic tumors through the induction of excessive reactive nitrogen species, such as nitrites, which induce massive DNA damage [224]. Importantly, in retrospective studies, the prognosis of surgically resectable BRCA-associated PDACs was like that observed for non-BRCA-mutated PDACs [225]. Some individual cases of BRCA-mutated PDACs treated with platinum and PARP inhibitor-based therapy were reported in the literature, usually showing an initial exceptional response to therapy and often a disease recurrence due to secondary BRCA mutations [226]. In addition to DDR deficiency mutations, there is also evidence that mutations in chromatin remodeling pathways, such as ARID1A mutations, could be targeted using PARP or ATR inhibitors [227]. ARID1A mutations are associated with the squamous PDAC subset associated with a negative prognosis, and ATR inhibitors could offer a therapeutic option for these patients.

The studies performed in other cancers have shown that the identification of some specific genetic abnormalities was the main way for the identification of new valuable therapeutic strategies. However, in many circumstances, the identification of a given somatic genetic abnormality is not sufficient and does not represent the direct therapeutic target, but another gene whose expression is altered because of a specific deregulation caused by the first mutated gene may represent the therapeutic target. A recent interesting and therapeutically promising study performed in PDAC cells offers an example of this condition. *RNF43* is mutated in 5–10% of PDACs. RNF43 encodes a transmembrane protein exhibiting the function of ubiquitin E3 ligase and seems to act as a tumor suppressor. The N-terminal domains of RNF43 induce Frizzled ubiquitination and, through this mechanism, suppress the Wnt signaling cascade. Loss-of-function mutations of the *RNF43* gene promote Wnt signaling activity, stimulate cell proliferation and result in neoplastic transformation. In PDACs exhibiting low RNF43 expression, an increased level of expression of Frizzled receptors was observed [228]. Interestingly, Steinhart and coworkers performed a genetic screen with CRISP-Cas9 genome editing enabling high-resolution detection of genetic vulnerability in RNF43-mutated PDAC cells [229]. Using this approach, they demonstrated that these cells are dependent for their growth and survival on the Frizzled receptor 5 (FZD5). Antibodies blocking FZD5 inhibit the growth of RNF43-mutant PDAC cells [229]. These observations open the way to clinical studies of targeting therapy in this PDAC subset.

Another approach to try to improve the response of PDACs to treatments could derive from a better understanding of the mechanisms involved in drug resistance in these cancer cells. Gemcitabine, a deoxycytidine analog that inhibits DNA replication, is used as a single-agent chemotherapy for PDAC. Although this drug is still used in the treatment, its effectiveness is strongly limited by the development of frequent resistance. In the time, gemcitabine was replaced by a polychemotherapy approach FOLFIRINOX (fluorouracil, leucovorin, irinotecan and oxaliplatin), eliciting better clinical responses than gemcitabine (4–5-month survival benefit) [230]. However, this regimen was associated with a considerable toxicity and represents an alternative regimen for patients with Eastern Cooperative Oncology Group (ECOG) 0 or 1. A recent meta-analysis based on all the clinical studies, which involved the use of FOLFIRINOX regimen for the treatment of PDAC patients with locally advanced disuse, showed that patients treated with this regimen had a median overall survival of 24 months longer than that reported with gemcitabine monotherapy [231]. However, in more recent studies, gemcitabine was associated with nab-paclitaxel (nanoparticle albumin-bound paclitaxel) allowing a therapeutic response at least comparable to that induced by FOLFIRINOX [229]. In patients who have undergone complete macroscopic resection for PDAC, the best treatments consist either of gemcitabine alone or gemcitabine and capecitabine [232].

Recently, cisplatin was added to the nab-paclitaxel + gemcitabine regimen and evaluated in 25 stage IV PDAC patients, reporting 8% complete responses and 62.5% partial responses, with an overall survival of 16.5 months and 20% of patients alive at 24 months [233]. These results, although obtained in a small number of patients, are very encouraging, and this regimen is being evaluated also in neoadjuvant and adjuvant settings. Controlled clinical trials will be required to evaluate the clinical impact of this new, three-drug regimen. Interestingly, most responder patients to this treatment seem to be enriched in BRACA1 or BRCA2 genetic-mutation related PDACs [233].

In some recent clinical studies, a modified (reduced chemotherapy intensity) FOLFIRINOX (m-FOLFIRINOX) was associated with new anti-cancer drugs. Thus, a recent phase I study reported the first results obtained administrating m-FOLFIRIFOX together with CPI-613, a drug targeting the altered form of mitochondrial energy metabolism in tumor cells, with consequent induction of apoptosis [233]. The results obtained in the 18 metastatic PDAC patients treated with the maximum tolerated dose were that 61% achieved an objective response [233].

Recent studies have explored the mechanisms of resistance of PDAC cells to gemcitabine, showing that increased glycolytic flux leads to addiction in pancreatic cancer cells and a corresponding increase in pyrimidine biosynthesis to enhance the intrinsic levels of deoxycytidine triphosphate (dCTP); increased levels of dCTP determine the effective levels of gemcitabine by competition [234]. This metabolic reprogramming is mediated by MUC1-mediated stabilization of hypoxia inducible factor-1α (HIF-1α). Targeting of HIF-1α or de novo pyrimidine biosynthesis, together with gemcitabine administration exert a strong anti-tumor effect [234]. These observations indicate potential targets to improve the efficacy of gemcitabine in PDAC patients.

Another approach to try to improve the therapeutic response of PDAC patients is based on the hypothesis that agents targeting the tumor microenvironment could improve the response of PDAC cells to chemotherapy agents. Studies of intravital imaging using the Rho kinase inhibitor Fasudil showed that this drug modifies the PDAC tumor microenvironment and improved pancreatic cancer response to chemotherapy (gemcitabine/nab-paclitaxel) [235]. This is a promising area of active experimental and clinical investigation. In this context, promising results were obtained with CCR2-blocking agents and with hyaluronidase.

In PDACs, the CCL2-CXCR2 chemokine axis is involved in the recruitment of tumor-associated macrophages, whose migration in the tumor microenvironment contributes to the development of an immunosuppressive condition. This pathway has prognostic implications in PDAC and is involved in chemio-radioresistance of PDACs [236], and its blockade restores anti-tumor immunity in preclinical models. Given this background, the CCR2 inhibitor PF-04136309 was tested in preclinical studies in combination with FOLFIRINOX chemotherapy. In the context of a phase I study, it was shown that the drug combination of FOLFIRINOX and Pfizer (PF)-04136309 induced toxic events comparable to those elicited by FOLFIRINOX alone, but resulted in a higher frequency of objective responses, compared to the group of patients treated with FOLFIRINOX alone [237]. Phase 2 and phase 3 studies are in progress to evaluate the therapeutic impact of PF-04136309 in improving the response to FOLFIRINOX regimen.

One of the main components of the extracellular matrix of the PDAC microenvironment is hyaluronic acid (HA); this compound raises the pressure of the interstitial fluid of the tumor stroma and reduces drug delivery to malignant cells. These observations have led to testing the experimental use of hyaluronidase as a therapeutic agent, aiming to degrade HA and to normalize the pressure of interstitial fluid of stroma. Thus, a PEGylated recombinant human HA (PEGPH20) was produced, showing promising results in preclinical and phase I clinical studies [238]. A randomized phase II study evaluated the association of PEGPH20 and gemcitabine/nab-paclitaxel vs gemcitabine/nab-paclitaxel showing an improvement of progression-free survival among patients with high HA in the association arm. These observations were fundamental for the ongoing phase III study assessing PEGPH20 in PDAC patients.

Understanding pancreatic cancer metabolism is of fundamental importance not only for a better comprehension of the biology of this tumor, but also for the identification of new therapeutic strategies. Several approaches have been attempted to target PDAC cell metabolism, and various experimental evidence has shown the existence of the metabolic vulnerabilities of PDAC cells. Thus, the PDCA cell-intrinsic alterations in various metabolic pathways, such as glutaminolysis, glycolysis, mitochondrial metabolism and redox homeostasis, represent potential targets for the development of new therapies.

The studies carried out on inhibitors of glutaminolysis are particularly interesting because they indicate the enormous capacity of metabolic adaptation displayed by PDAc cells. The first step in the process of glutaminolysis is catalyzed by the enzyme glutaminase (GLS), responsible for the conversion of glutamine to glutamate in the mitochondria; in PDAC cells, glutamate in the mitochondria is metabolized, resulting in increasing the reducing potential in the form of increased NADPH and GSH [81]. The disruption of glutamine metabolism through inhibition of GLS results in increased antioxidant response and reduced cell growth. Glutaminase inhibitors are synergistic with metformin in inhibiting the growth of PDAC in various animal models [239]. Furthermore, glutaminase inhibitors sensitize pancreatic cancer cells to PARP-driven cytotoxic effects on PDAC cells [240]. However, the analysis of the behavior of PDAC cells exposed to GLS inhibitors showed that these cells, after an initial marked anti-proliferative response, display a progressive metabolic adaptation, activating compensatory metabolic networks that sustain proliferation in vitro and in vivo [241]. Therefore, the combined targeting of glutamine metabolism and of these adaptive pathways is required to yield the expected clinical benefits for PDAc patients [241].

The numerous abnormalities of the glycolytic pathway present in PDAc cells have been characterized in the last few years. Pancreatic cancer cells are characterized by a high increase in glucose uptake and metabolism and high glycolytic rates (Warburg effect) [93]. Numerous other studies have confirmed this activation of oxidative glycolysis in PDAC (reviewed in [242]). In fact, a significant overexpression of glycolytic enzymes and lactate dehydrogenase was observed, in line with the Warburg effect, to promote rapid adenosine-triphosphate (ATP) production from glycolysis [242]. Various overexpressed glycolytic enzymes were identified as potential therapeutic targets [242]. At variance with the observations on glycolysis, the Warburg effect on mitochondrial oxidative phosphorylation enzymes were less clear, suggesting that other metabolic pathways are stimulated in PDAC cells [242]. Some recent studies have tried to explore the mechanism of enhanced glycolysis in PDACs. Nagarajan and coworkers have developed an integrative genomic approach to identify deregulated components of the glycolytic apparatus in PDAC; using this approach, they have identified a pathway involving paraoxonase 2 (PON2) as a regulator of the glucose transporter 1 (GLUT1) and consequential activation of the AMP-activated protein kinase (AMPK) → forkhead box 3 (FOXO3A) → p53-upregulated modulator of apoptosis (PUMA) pathway [242]. In PDAC cells, PON2 is overexpressed and activates GLUT1 via stomatin [243]. The loss or inhibition of PON2 activates a starvation pathway, leading to AMPK activation, PUMA activation and anoikis and inhibition of tumor cell growth [242]. Importantly, AMPK activation exerts effects like PON2 inhibition [243].

The enhanced glycolytic activity of PDAc cells is sustained also through a mechanism involving KRAS and p16-mediated activation of NOX4: this enzyme catalyzes NADH oxidation and, through this mechanism, supports glycolysis by generating NAD^+^, a substrate for GAPDH-mediated glycolytic reaction [97]. A peculiar abnormality of the glycolytic metabolism was recently reported in PDAC cells displaying *SMAD4* locus deletion. In fact, SMAD4 deletion determines co-deletion of the Mal-2 gene locus [12]. The family of malic enzymes includes three enzyme isoforms: a cytosolic NADP^+^-dependent isoform ME1 and two redundant mitochondrial NAD(P)^+^-dependent isoforms (ME2 and ME3). In normal conditions, ME2 is the predominant isoform; however, in the eventuality of ME2 deficiency, ME3 activity replaces ME2 deficiency, providing NADPH required for mitochondria. ME3 depletion induces the killing of ME2-null PDAC cells [12]. Thus, this study has shown a vulnerability of SMAD4-mutated PDAC cells.

Other studies have linked changes in glycolysis with the PDAC metastatic process. A large-scale reprogramming of chromatin modifications was observed in PDAC cells during their metastatic evolution [62]. These changes occur in association with the development of a dependence on the oxidative branch of the pentose phosphate pathway (oxPPP); importantly, oxPPP inhibition reversed reprogrammed chromatin, malignant gene expression programs and tumorigenesis [62].

Other observations have shown a main role of liver kinase B1 (LKB1) deregulation in the modifications of cellular metabolism observed in PDAC. LKB1 is a key regulatory protein of cellular metabolism, originally identified in patients with Peutz-Jeghers syndrome, an autosomal dominant disease with LKB1 germline mutations. LKB1 acts via the AMP-activated protein kinase (AMPK) pathway. LKB1 is mutated in about 2% of PDACs [244]. LKB1 protein levels are clearly decreased in about 50% of PDACs, and patients with low LKB1 levels have a negative prognosis [245]. Inactivation of LKB1 results in the activation of the mammalian target of rapamycin (mTOR) pathway, which plays a key role in the control of cell energetic metabolism, cell survival and growth under metabolic stress, such as nutrient deficiency. Given the peculiar microenvironment in which pancreatic cancer cells grow, characterized by the desmoplastic response and by a reduced blood supply to tumor cells, it is evident that LKB1 inactivation may play a tumorigenic role in PDAC development. In line with these observations, LKB1 haploinsufficiency cooperates with KRAS in promoting pancreatic cancer development though suppression of p21-dependent growth arrest [246]. A recent study better clarified the role of LKB1 in promoting pancreatic cancer development showing that the oncogenic cooperation between LKB1 loss and KRAS activation is mediated by pronounced mTOR activation, which in turn activates the serine-glycine-one-carbon pathway coupled to S-adenosylmethionine generation; in parallel, DNA methyltransferases are upregulated, leading to elevation in DNA methylation, particularly at the level of retrotransposon elements, with their consequent silencing [246]. Importantly, this pathway triggered by LKB1 deficiency exposes PDAC cells to a potential vulnerability to inhibition of serine biosynthesis and DNA demethylation [247]. Dietary serine starvation is an experimental strategy to reduce the growth of some tumors [248]; unfortunately, KRAS-driven mouse models of pancreatic cancer are less responsive to depletion of serine, reflecting the ability of activated KRAS to increase the expression of enzymes involved in serine biosynthesis [248].

The study of some animal models of pancreatic cancer has permitted the identification of new potential therapeutic targets for PDAC. In this context, particularly interesting were the results of a study by Genovese and coworkers, based on the study of cancer cell plasticity in a conditional oncogenic KRAS model of PDAC [249]. In this model, stochastic extinction of KRAS signaling was associated in the escaper populations with the development of de-differentiated tumors, with aggressive biological behavior; these tumors displayed a mesenchymal reprogramming [248]. This reprogramming was based on the activation of a Myc-dependent network, triggering an anabolic switch that increases protein metabolism and adaptive activation of endoplasmic reticulum stress-induced survival pathway [249]. Interestingly, this metabolic adaptation renders PDAC cells highly susceptible to pharmacological inhibition of the cellular proteostatic machinery and the IRE1-α-MKK4 arm of the endoplasmic-reticulum-stress-response pathway [249]. These observations suggest a potential therapeutic strategy for targeting aggressive mesenchymal features of PDACs [249].

The field of immunotherapy has recently generated interesting observations in some tumors, including melanomas and lung cancers. Particularly, the use of checkpoint inhibitors, such as those blocking programmed cell death 1 (PD-1), PD-ligand 1 (PD-L1) and cytotoxic T lymphocyte-associated protein 4 (CTLA-4) has shown significant therapeutic responses in these tumors. Unfortunately, clinical studies carried out using these drugs in PDAC patients have failed to achieve any significant clinical response, a phenomenon seemingly related to the high immunosuppressive activity of PDAC [250]. The only encouraging results were observed in a cohort of patients with PDAC with mismatch repair-deficient tumors [251]. This last finding is not surprising in that a recent study exploring mutational signatures in large cohorts of PDAC patients (including a retrospective cohort, a discovery cohort and a replication cohort of PDAC patients) showed the existence of four major subtypes; interestingly, mechanisms of both germline and somatic genomic instability, typical of DNA mismatch repair and double-stranded break repair, were observed in 12% of cases and were associated with transcriptional and immunohistochemical markers of immune activation [252].

Given the overall very limited responses of single-agent checkpoint inhibition and supporting some evidence coming from pre-clinical studies, the strategy of immunotherapy studies in PDAC has shifted to various types of combination therapies involving either dual checkpoint blockade, the combination of immunotherapy with chemotherapy, targeted therapeutics or radiation.

At the end of this brief outline of the identification of some possible new therapies for PDAC, it is particularly interesting to analyze and discuss the negative results of a recent trial of targeted therapy in PDAC patients. This trial was based on the randomized comparison of gemcitabine plus vandetanib versus gemcitabine plus placebo in PDAC patients with metastatic or locally advanced disease [253]. Vandetanib is a multi-tyrosine-kinase inhibitor targeting VEGFR2, EGFR and RET. Unfortunately, the results of this trial were negative with a median overall survival of 8.83 months in the gemcitabine plus vandetanib group, compared to 8.95 months in the group with gemcitabine plus placebo [254]. Thus, this trial is an additional example of a negative trial of targeted therapies in PDAC. The main reason why these trials have failed in PDAC is seemingly related to the presence in this tumor of numerous redundant oncogenic signaling pathways, with an extensive level of crosstalk. However, a great contribution to the failure of some of these studies was originated from an inappropriate design and, particularly, from the absence of valuable biomarkers. Since PDAC is a highly heterogeneous tumor at the molecular level, the identification of a subgroup of patients who might benefit from a given, specific therapy is mandatory. These observations imply that well designed trials, including comprehensive translational programs focused on identifying predictive biomarkers for molecularly defined subgroups, must be performed.

## Figures and Tables

**Table 1:** Histological and molecular properties of the main types of pancreatic tumors. The most recurrent genetic alterations observed in PDAC are marked in bold.

Tumor Type	Prevalence (% of Pancreatic Tumors)	Cell Differentiation	Histopathology	Immunohistochemical Markers	Average of Somatic Mutations/Tumor	Recurrent Genetic Alterations
Pancreatic Ductal Adenocarcinoma (PDAC)	90%	Ductal	Ductal and glandular structures. Presence of abundant desmoplastic stroma. Blood vessels, lymphatic and perineural tumor invasion. Presence of cellular elements with enlarged pleomorphic nuclei and eosinophilic cytoplasm.	SMAD4 loss Aberrant TP53 expression Expression of several mucins, including MUC1, MUC3, MUC4 and MUC5AC.	20–80	**KRAS** **SMAD4** **TP53** **CDKN2A** **KDM6A** MLL3, TGFBR2, ATM, ARID1A, ROBO1, ROBO2
Pancreatic Neuroendocrine Tumors (PanNET) Pancreatic Neuroendocrine Cancer (PanNEC) Familial Sporadic	5%	Neuroendocrine	PanNET is characterized by the presence of nests of cells or cords; a key feature is represented as a homogenous cell population with a stippled chromatin (“salt and pepper” nuclei)	Expression of neuroendocrine markers: synaptophysin and chromogranin, peptide hormones, such insulin and glucagon. For PanNECs: aberrant nuclear expression of TP53.	16	Sporadic PanNET: MEN1, ATRX or DAXX, VHL and PHLDA3 deletion. ATRX or DAXX loss is associated with The alternative lengthening of telomeres. Familial PanNET: MEN1. PanNECs: KRAS, TP53 and RB1.
Pancreatic Acinar Cell Carcinoma (PACC)	1–2%	Acinar	Presence of acinar units. Cellular elements with enlarged nuclei with prominent nucleoli and with finely granular cytoplasm.	Pancreatic exocrine enzymes: trypsin, chymotrypsin, lipase. Immunoreactivity with anti-BCL10 mAb, due to homology with CEL protein present in acinar cells. Expression of PDX1.	20–149	SMAD4, TP53, APC, BRAF, BRCA2, RB1, ARID1A, GNAS, MLL3, PTEN
Solid-Pseudopapillary Neoplasm	1–2%	Acinar	Uniform population of poorly cohesive cells forming solid or pseudopapillary structures. Cells with round nuclei, eosinophilic or clear cytoplasm.	Nuclear labeling of β-catenin. Expression of CD10. Paranuclear labeling of CD99 and of LEF-1 (lymphoid-enhancer binding factor-1). Loss of membranous E-cadherin.	3	CTNBB1 exon 3 mutation (90% of cases).
Pancreatoblastoma	<1% (adult) 25% (pediatric)	Acinar	Notes of acinar differentiation. Typical squamoid nests. Presence of neuroendocrine and ductal elements.	Pancreatic exocrine enzymes. Nuclear labeling of β-catenin. SMAD4 loss.	18	CTNNB1 Loss of chromosome 11p

**Table 2:** Main medical treatments for advanced stage PDAC.

Drug Type/Target	Clinical Studies
Cytotoxic Chemotherapy Gemcitabine Combinations	Multiple phase II and III studies have been evaluated. Only erlotinib (EGFR inhibitor) very slightly improved the survival, compared to Gemcitabine alone. Nab-paclitaxel (albumin-bound formulation of paclitaxel) in association with gemcitabine resulted in a 2-month survival benefit. A phase Ib/II pilot trial evaluated nab-paclitaxel plus gemcitabine plus cisplatin in patients with stage IV pancreatic cancer, resulting in a high rate of clinical responses. The European Society Pancreatic Adenocarcinoma (ESPAC)-4 trial, a randomized phase III study, compared the adjuvant administration to surgically-resected PDACs of gemcitabine plus capecitabine with gemcitabine monotherapy: the median overall survival of the combined regimen was 28 months, compared to 25.5 months for the monotherapy regimen. A pilot phase Ib/II pilot trial (NCT01893801) evaluated the safety and the clinical efficacy of a regimen based on gemcitabine, plus nab-paclitaxel, plus cisplatin in patients with stage IV PDAC, reporting 8% complete responses, 62.5% partial responses and 16.5% stable disease. This regimen is under evaluation in patients in stage IV PDAC patients, neoadjuvant and adjuvant settings. The NCT02551991 trial is comparatively evaluating non-liposomal irinotecan-containing regimes versus nab-paclitaxel, plus gemcitabine in patients with previously untreated metastatic pancreatic adenocarcinoma.
Cytotoxic Chemotherapy FOLFIRINOX	FOLFIRINOX is an alternative regimen (fluorouracil, irinotecan, oxaliplatin) for PDAC patients with Eastern Cooperative Oncology Group (ECOG) 0 or 1. Compared to gemcitabine monotherapy, it gives a 4-month survival benefit. Modified FOLFIRINOX (mFOLFIRINOX) is a reduced-intensity regimen, often used in association with some targeting agents.
Molecular Targeting BRCA	The NCT02184195, a phase III, randomized, double-blind, placebo-controlled, multicenter study of maintenance olaparib monotherapy in patients with BRCA-mutated metastatic pancreatic cancer whose disease has not progressed on first line platinum-based chemotherapy.
Mitochondrial Targeting CPI-613	The NCT0183504 trial is a phase I study evaluating CPI-613 and combination chemotherapy (mFOLFIRINOX) in patients with metastatic pancreatic cancer. Of the 18 treated patients given the Maximum Tolerated Dose (MTD), 61% achieved an objective (complete or partial) response.
Microenvironment Targeting Tumor-Associated-Macrophages CCL2/CCR2 Axis	The NCT01413022 trial, a phase Ib study, evaluated targeting of tumor-associated macrophages with CCR2 inhibition (PF-04136309) in combination with FOLFIRINOX in patients with borderline resectable and locally advanced pancreatic cancer; 49% of treated patients achieved an objective response. The NCT 0235408 trial, a phase Ib study, evaluated a CCR2 selective inhibitor (CCX872-b) in association with FOLFIRINOX in patients with metastatic pancreatic cancer; at 12-week post-treatment tumor control was observed in 78% of treated patients, with 37% partial responders and 41% with stable disease.
Microenvironment Targeting Tumor-Associated-Macrophages CSF1/CSF1R Axis	The NCT 02777710 trial, a dose-escalation phase I study, with an extension part evaluating the safety and activity of an anti-PD-L1 antibody (durvalumab) combined with a small molecule CSF-1R tyrosine kinase inhibitor (pexidartinib) in patients with metastatic advanced pancreatic or colorectal cancers.
Microenvironment Targeting Extracellular Matrix Hyaluronic Acid	The NCT02715804 trial, a phase III, randomized, double-blind, placebo-controlled, multicenter study of PEGylated recombinant human hyaluronidase (Stoma-PEGylated Recombinant Human Hyaluronidase 20 (PEGPH20)) in combination with nab-paclitaxel and gemcitabine in participants with hyaluronan-high stage IV previously untreated pancreatic ductal adenocarcinoma; 45% of patients in the PEGPH20 plus chemotherapy reported an objective response, compared to 31% in patients receiving chemotherapy alone.
Microenvironment Targeting CXCL12/CXCR4 Axis	The NCT 02826486 trial, a phase II, multicenter, open-label single arm study to assess the safety and efficacy of the combination of BL-8040 and pembrolizumab in patients with metastatic pancreatic cancer, the COMBAT study/KEYNOTE-202.
